# Protein tyrosine kinase-6 is post-translationally regulated by arginine methylation and ubiquitination

**DOI:** 10.1038/s41419-026-08933-5

**Published:** 2026-06-05

**Authors:** Liping Jiang, Milica B. Gilic, Katarina Vlajic, Wenjun Bie, Shafi Kuchay, Angela L. Tyner

**Affiliations:** 1 Department of Biochemistry and Molecular Genetics, University of Illinois at Chicago, Chicago, IL, USA; 2 University of Illinois Cancer Center, University of Illinois at Chicago, Chicago, IL, USA; 3 Present Address: University of Washington, Seattle, WA USA

**Keywords:** Kinases, Methylation, Ubiquitylation, Oncogenesis

## Abstract

Protein tyrosine kinase 6 (PTK6) has context-specific, kinase-dependent and -independent functions in normal cells and cancer. In prostate cells, nuclear PTK6 suppresses growth, while active membrane-associated PTK6 promotes oncogenic signaling. Unlike SRC-family kinases, PTK6 is not modified by lipidation to promote membrane association. Although arginine residues in the PTK6 SH2 domain were previously linked to lipid binding, we found they are instead critical for protein stability and arginine methylation. Mutation of R131/R136 or R132 impaired PTK6 ubiquitination and its association with a Cullin-1-RING ubiquitin ligase complex, and its half-life increased from one hour to over 24 h. We show that PTK6 is targeted by PRMT1 and mutation of PTK6 R131/R136 or R132, or treatment with the type 1 PRMT inhibitor GSK3368715, leads to decreased arginine methylation, increased PTK6 protein stability and impaired PTK6 association with its substrates. In prostate cancer cells, ectopic expression of mutant PTK6 R131/R136 or R132 leads to reduced proliferation, anchorage-independent growth, and cell migration and invasion, when compared with wild type PTK6, even though higher levels of mutant protein are expressed. These results indicate that arginine methylation is a key regulatory switch for PTK6 signaling and protein turnover and may be central for the execution of its diverse context-specific functions. Mutation of PTK6 arginine residues has been reported in some cancers, which may contribute to disease-specific PTK6 expression and signaling.

## Introduction

PTK6 (also called BRK) is an intracellular tyrosine kinase that is distantly related to the SRC-family. Unlike SRC family members, PTK6 lacks membrane-targeting lipid modifications; it is not myristoylated or palmitoylated. It also lacks a conventional nuclear localization signal. However, PTK6 localizes to different cellular compartments including the plasma membrane and nucleus, depending on tissue and cell type, and its intracellular localization dictates its functions (reviewed in [1–3]).

PTK6 is concentrated in the nucleus in the normal prostate but it is found in the cytoplasm and at the plasma membrane in prostate cancer [4]. Re-localization of PTK6 out of the nucleus promotes its oncogenic signaling [5–7]. Lipid modification plays an important role in membrane localization and transformation by SRC-family kinases [8]. Targeting PTK6 to the plasma membrane by addition of the palmitoylation/myristoylation signal from the LYN tyrosine kinase was sufficient to transform immortalized mouse embryonic fibroblasts, deficient for SRC, YES, and FYN [9], and for induction of the epithelial-mesenchymal transition in prostate cancer cells [10]. PTK6 activation and membrane association has been detected in mouse and human prostate [10, 11], breast [12], and skin [13] tumors.

Previously, Park and colleagues identified the PTK6 SH2 domain in a screen for SH2 domains that bind to phospholipids [14]. Arginine residues [R] 131 and 136 located within the PTK6 SH2 domain were implicated in lipid binding and membrane association. It is recognized that membrane association of PTK6 could also be controlled through protein-protein interactions at the plasma membrane, and histidine [H] 126 is involved in binding phosphotyrosine (PY) residues in associated proteins [15]. In our studies, lipid binding and membrane association is retained upon mutation of R131A/R136A or R132A, while mutation of H126 leads to decreased PTK6 membrane localization.

Here we determined that arginine residues 131, 132, and 136 in the SH2 domain are methylated and important for regulation of PTK6 protein stability via a Cullin 1-ring ubiquitin ligase complex. Methylation of arginine residues within a variety of protein substrates regulates many cellular functions including signal transduction, transcription and RNA metabolism [16, 17]. We show that PTK6 is targeted by protein arginine methyl transferase 1 (PRMT1), and this has an impact on its activities and ubiquitination. Although mutation of arginine residues impairs PTK6 turnover, it also reduces PTK6 oncogenic functions. Understanding the mechanisms and significance of PTK6 post-translational modifications may provide novel insights about PTK6 functional regulation and expose new avenues for targeting its oncogenic functions.

## Materials and methods

### Cell lines and treatments

PC3 (CRL-1435), DU-145 (HTB-81), and LNCaP (CRL-1740), and HEK293 (CRL-1573) cells (ATCC), and BPH1 cells (gift from Simon Hayward) were used. Transfections were performed using Lipofectamine 2000 (Thermo Fisher Scientific, Waltham, MA, USA) according to manufacturer’s instructions. For inhibitor assays, LNCaP and BPH1 cells were treated with 10 μM MLN4924 (B1036, ApexBio) or 10 μM MG132 for (0–8 h) or varying doses of GSK3368715 (MedChemExpress, Monmouth Junction, NJ) (2 μM–10 μM for 24 h and 48 h) prior to lysis in Triton-X 100 buffer.

### Expression constructs

N-terminal MYC-tagged PTK6 constructs, including wild type (WT), R132A, R131A/R136A, and R131A/R132A/R136A, constitutively active PTK6 YF, kinase dead (K219M, KM/R131A/R136A), pY binding mutant H126A, were cloned into pcDNA3. GFP-tagged human wild-type PTK6 was cloned into pEGFPc1. GFP-tagged YES1, LYN, FYN, and SRC, and FLAG-TR-Tube constructs (wild type and mutant) have been described [18]. pcDNA3-DN-hCUL1-FLAG (#15818), pcDNA3-DN-hCUL2-FLAG (#15819), pcDNA3-DN-hCUL3-FLAG (#15820), pcDNA3-DN-hCUL4A-FLAG (#15821), pcDNA3-DN-hCUL4B-FLAG (#15822), pcDNA3-DN-hCUL5-FLAG (#15823) were gifts from Wade Harper (Addgene plasmids). MYC-tagged PTK6 constructs (PTK6 WT, PTK6 R132A, and PTK6 R131A/R136A were cloned into the pBabe-puro vector. PRMTs 1–9 were cloned into pEGFPc1 vector using different restriction sites.

### Protein lysates, cell fractionation, and immunoblotting

Cells were given fresh media 10 min prior to harvesting. Total cell lysates were prepared and subjected to immunoblotting as previously described [6]. Cell fractionation was performed using the ProteoExtract subcellular proteome extraction kit (Calbiochem, Billerica, MA) as described in manufacturer’s instructions. Immunoprecipitation was performed as previously published [6]. RIPA lysis buffer containing 50 mM Tris-HCl, pH 7.6, 150 mM NaCl, 1% NP-40, 1% sodium deoxycholate, 0.1% SDS, 1 mM EDTA, 1 mM PMSF was used for protein isolation for lipid dot blots.

### Antibodies

Antibodies were purchased from the following sources, Santa Cruz Biotechnology (Dallas, TX): monoclonal PTK6 (BRK G-6, sc-166171, 1:1000 for immunoblotting (IB), 1:100 for immunofluorescence (IF); PY (sc-508, 1: 1000); FAK (sc-558, 1: 2000). Millipore (Billerica, MA): SRC (05-184, 1: 2000); PTK6 PY342 (09-114, 1:1000 for IB, 1:100 for IF); Ubiquitin (sc-166553, 1: 1000); PY (05-321, 1: 1000). Abcam (Cambridge, MA): PTK6 PY447 (ab138368, 1:1000 for IB, 1:100 for IF); Mono and dimethyl Arginine antibody (ab412, 1:2000). BD Biosciences (San Jose, CA): p27 (554069, 1:1000); β-catenin (610154, 1:2000). Cell Signaling Technology (Danvers, MA): CUL1 (4995, 1: 1000); GAPDH (2118, 1:10000); GFP (2956, 1: 2000); MYC-tag (2276, 1: 2000, for IB, 1: 200 for IF); SKP1 (12248, 1: 1000); K48 polyubiquitin (8081S, 1: 2000); STAT3 (9139S, 1:2000); PY705-STAT3 (9145S, 1:2000). Sigma-Aldrich (St. Louis, MO): α-tubulin (T-9026, 1: 2000); FLAG-tag (F3165, 1: 2000); AKT (9272, 1: 2000); PS473-AKT (9271, 1: 2000); PY925-FAK (3284, 1: 2000); PY416-SRC (2101, 1: 2000); EGFR (2232, 1: 2000); PY845-EGFR (2231, 1: 2000). Covance (Cumberland, VA): GST tag (4C10, MMS-112R, 1:1,000). Zymed (San Francisco, CA): His-tag (37-2500, 1: 2000). Donkey anti-rabbit (NA934, 1:500) and sheep anti-mouse (NA931, 1:500) antibodies conjugated to horseradish peroxidase were purchased from GE Healthcare (Pittsburgh, PA) and detected by chemiluminescence with SuperSignal West Dura substrate (34075) from Thermo Fisher Scientific (Waltham, MA).

### Immunofluorescence

Cells were cultured on 8-well chamber slides (ThermoFisher Scientific) and stimulated with fresh growth media for 10 min at 24 h post-transfection and then fixed in 4% PFA. Full-length MYC-tagged wild-type and mutant PTK6 were transiently co-expressed with td-Tomato PMV-SeQ [19]. Primary antibodies were detected using biotinylated goat anti-rabbit and streptavidin-conjugated Alexa Fluor 488 purchased from Invitrogen (Waltham, MA), and localization was assessed by immunofluorescence using ZEN software (Zeiss, Jena, Germany).

### Lipid binding

PIP strips were obtained from Echalon Biosciences. Lipids spotted on membrane strips included eight different phosphoinositides (PI: phosphatidylinositol; PI(3)P: phosphatidylinositol (3)-phosphate; PI(4)P: phosphatidylinositol (4)-phosphate; PI(5)P: phosphatidylinositol (5)-phosphate; PI(3,4)P2: phosphatidylinositol (3,4)-bisphosphate;PI(3,5)P2- phosphatidylinositol (3,5)-bisphosphate; PI(4,4)P2: phosphatidylinositol (4,4)-bisphosphate; and PIP3: phosphatidylinositol (3,4,5)-trisphosphate. Seven additional lipids are also included on the strips, including LPA: lysophosphatidic acid; LPC: lysophosphocholine; PE: phophatidylethanolamine; PC: phosphatidylcholine; SIP: sphingosine 1-phosphate; PA: phosphatic acid; and PS: phosphatidylserine. Strips were blocked with 3% fatty acid free BSA (Sigma-Aldrich, St. Louis, MO, USA) in TBST for 1 h at room temperature. Transfected HEK293T cells were lysed in RIPA lysis buffer containing protease inhibitors. Lysates were pre-cleared using Protein G Agarose (Invitrogen) by rotating for 1–2 h. Immunoprecipitations were performed using anti-MYC agarose (Sigma-Aldrich), and immunoprecipitated proteins were washed and incubated with the strips according to instructions.

### Half-life measurement

At 24 h post-transfection, regular growth media was changed to growth media with 100 μM of cycloheximide (CHX, Sigma-Aldrich) for 0, 0.5, 1, 2, 4, 8, 12, 24 h and harvested. Lysates were subjected to immunoblotting and bands for MYC-tagged proteins were scanned, quantified, and normalized. The protein half-life was calculated using GraphPad Prism (nonlinear regression, one-phase decay).

### Inhibition of proteasomal degradation and detection of ubiquitination

At 20 h post transfection, regular growth media was changed to growth media with 10 μM of MG-132 (Sigma-Aldrich) and cells were harvested after four hours. Immunoprecipitation of FLAG-TR-Tube was performed with FLAG M2 agarose, followed by immunoblotting with PTK6 antibody for detection of ubiquitinated PTK6.

### Biological assays

To generate stable LNCaP cell pools PTK6 constructs were expressed via p-Babe-puro retroviral infection and selected with 2 μg/ml puromycin. For proliferation assays, 1 × 10^4^ cells were monitored at indicated time points and normalized to the 0 h time point. For colony formation assays, cells were seeded in triplicate at a density of 1 × 10^3^ cells per well of six-well plates, and grown for 14 days, and then fixed with 4% PFA and stained with 0.5% crystal violet (Sigma-Aldrich). For soft agar assays, 1.5 × 10^4^ cells were seeded on a 0.7%/0.45 agar bilayer and counted at four weeks post-plating. For migration/invasion, transwell assays (8 μm pore; Corning) measured migration toward 20% FBS at 24 h, or invasion through Matrigel (diluted 1:3 in serum-free medium) at 48 h. Cells on the lower surface of transwell membranes were stained by crystal violet and images were taken at 20 X magnification. All experiments were performed in triplicate and repeated independently at least three times.

### Quantification and statistics

Quantification of immunoblots was performed using ImageStudio Lite (LI-COR Biosciences, Lincoln, NE) or image J. For all experiments, at least 3 independent trials were performed. *P*-values were determined using a two-tailed, unpaired Student’s t-test (GraphPad Prism, San Diego, CA). A difference was considered statistically significant if the *p*-value was less than 0.05. Significance was defined as ns = *p* > 0.05, * < = *p* % 0.05, ** < = *p* % 0.01, *** < = *p* % 0.001, **** < = *p* % 0.0001. Error bars represent mean ± the standard error of the mean (SEM). Statistics were calculated by Student’s t-test, one-way ANOVA followed by Tukey’s test or paired two-way ANOVA with multiple comparisons, as indicated.

## Results

### Arginine residues 131, 132 and 136 in the SH2 domain are dispensable for PTK6 binding to membrane lipids

**Fig. 1:**
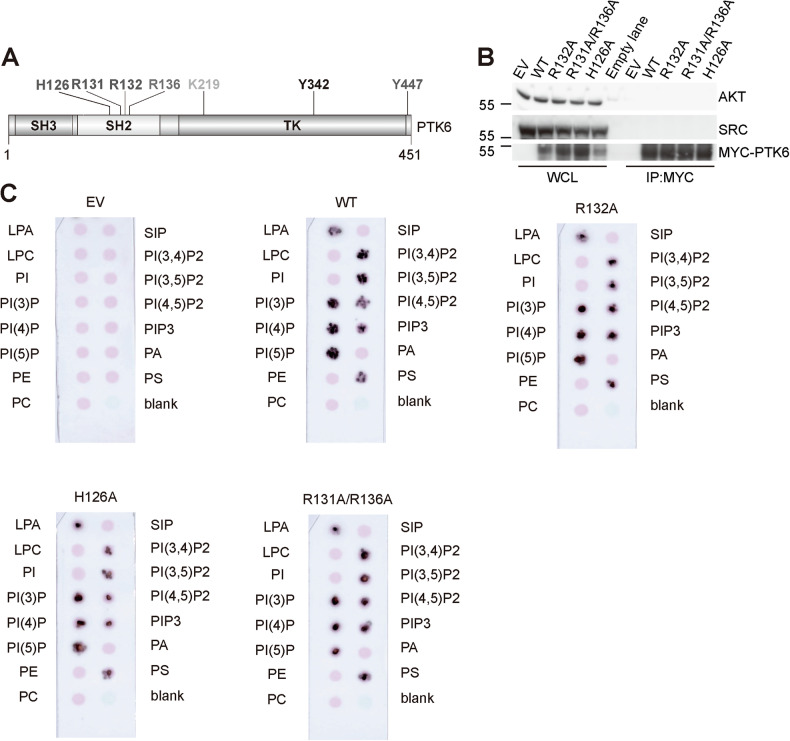
PTK6 SH2 domain mutants associate with lipids like the wild type PTK6 protein. **A** Schematic representation of PTK6 protein domains highlighting key residues: H126 (phosphotyrosine binding), putative lipid binding sites R131, R132, and R136, K219 (ATP binding), Y342 (activation loop), and Y447 (C-terminal regulatory tyrosine). **B** MYC-tagged PTK6 (WT or mutants H126A, R132A, and R131A/R136A), or empty vector (EV) were transfected into HEK293T cells. Immunoprecipitated MYC-tagged proteins were probed for coimmunoprecipitation of MYC-PTK6 with its substrates AKT and SRC. No PTK6 associated proteins were detected under the conditions used. **C** MYC-tagged PTK6 WT or MYC-PTK6 mutants were immunoprecipitated from HEK293T cells and used as probes in protein-lipid overlay assays.

Although it is not myristoylated or palmitoylated, PTK6 shares structural homology with SRC family kinases (Fig. 1A). It has a short unique amino terminus followed by SH3, SH2, and kinase catalytic domains. Like SRC family kinases, PTK6 activity is negatively regulated by intramolecular interactions mediated by phosphorylation of a carboxy terminal tyrosine residue at amino acid position 447 [20]. Phosphorylation of tyrosine residue 342 within the catalytic domain corresponds with PTK6 kinase activation [11, 20]. Mutation of lysine residue 219, which is in the PTK6 catalytic domain and is involved in ATP binding, renders the kinase inactive [21]. Amino acid residue H126 within its SH2 domain is involved in PTK6 binding to phosphotyrosines in associated proteins [15]. The PTK6 SH2 domain was identified in a screen for SH2 domains that bind phospholipids, and amino acid residues R131 and R136 were identified as important for lipid-binding [14].

We tested the ability of PTK6 to bind different lipids in a protein-lipid dot blot overlay assay. We expressed MYC-tagged full-length wild-type (WT) PTK6 or full-length mutant PTK6 H126A, R131A/R136A, or R132A in HEK293 cells, and immunoprecipitated the MYC-tagged proteins under stringent conditions that do not facilitate protein-protein association. As a control, we probed immunoprecipitated protein for PTK6-associated proteins that bind phospholipids, AKT [22] and SRC kinase [23], and these PTK6 substrates did not coimmunoprecipitate (Fig. 1B). Immunoprecipitated PTK6 proteins were used to probe PIP strip membranes, hydrophobic membranes spotted with 100 pmol of fifteen different biologically relevant lipids (Fig. 1C). The protein lipid overlay assay allows testing for lipid binding specificity and relative affinity of protein samples for a range of biologically relevant lipids in a single experiment, but results are qualitative, and association constants cannot be determined using this method [24]. Similar patterns of lipid binding were detected for wild-type PTK6 and all mutant PTK6 proteins.

We also probed PIP strips with purified recombinant GST-tagged full-length wild-type or kinase-dead PTK6 (PTK6 KM), or wild type and mutant GST-tagged PTK6 SH2 domains produced in bacteria for the assays (Fig. S1A, B). We confirmed purity and activity of the full-length GST-tagged PTK6 variants by immunoblotting for GST and phosphotyrosines (PY) (PY342 and total PY) (Fig. S1C). Neither activity of the full-length protein (Fig. S1D, Fig. S2), nor mutation of specific residues in the SH2 domain (Fig. S1E) had a major impact on lipid binding in these in vitro assays.

### PTK6 membrane localization is retained despite mutation of previously identified lipid binding arginine residues in SH2 domain

**Fig. 2:**
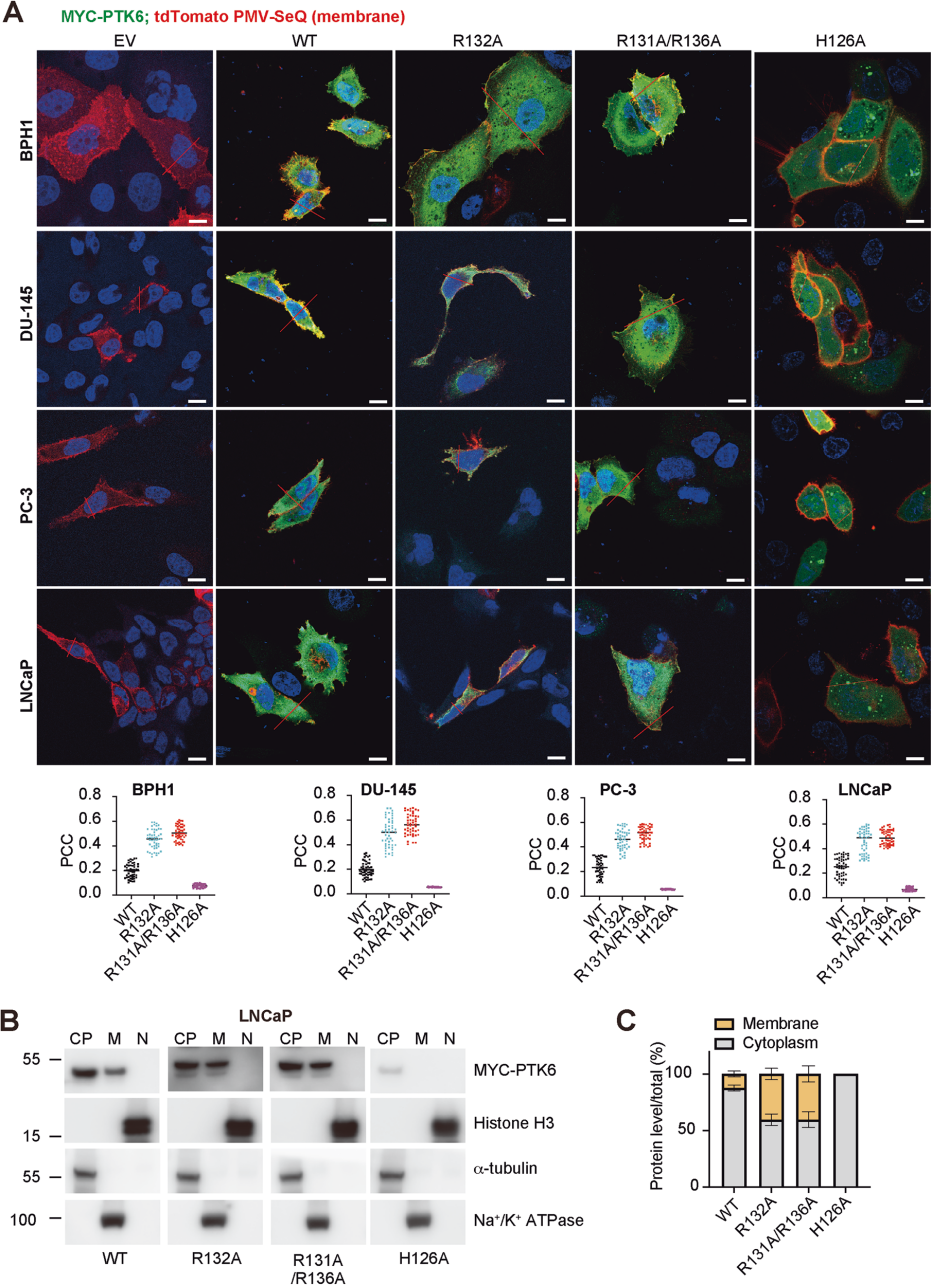
Mutation of arginine residues R132 or R131/R136 does not reduce membrane association of PTK6 in prostate cell lines. **A** Prostate cell lines BPH1, DU-145, PC3, and LNCaP were transfected with empty vector (EV) or MYC-tagged PTK6 WT, R132A, R131A/R136A, or H126A along with tdTomato-tagged PMV-SeQ (plasma membrane marker) and localization of the ectopic proteins was analyzed by immunofluorescence. MYC-tagged PTK6 was detected with MYC-tag antibody. Nuclei were counterstained with DAPI. Scale bar: 10 μm. Confocal imaging is shown by representative captured images. Colocalization of MYC-tag (green) and PMV-SeQ (red) in each cell line was quantified using Pearson correlation coefficients (PCC), *N* = 50 cells. Statistics were calculated by one-way ANOVA followed by Tukey’s test. Error bars indicate SEM. Analysis of colocalization of green and red fluorescence is shown in Fig. S3. **B** LNCaP cells expressing MYC-PTK6 WT or MYC-PTK6 mutants were fractionated into cytoplasmic (CP), membrane/organelle (M), and nuclear (N) fractions. MYC-PTK6 was detected with MYC-tag antibody. Fractionation controls: Na⁺/K⁺ ATPase (plasma membrane), α-tubulin (cytoplasm), Histone H3 (nucleus). Experiments were repeated at least three times independently. **C** Quantification of the relative distribution of wild type and mutant PTK6 in the cytoplasmic and membrane fractions is shown, calculated as percentage of total signal detected in each fraction. Statistics were calculated by one-way ANOVA followed by Tukey’s test, *N* = 3 independent experiments.

We investigated if SH2 domain mutations (H126A, R131A/R136A, R132 A) alter PTK6 intracellular localization in prostate cell lines. Full-length MYC-tagged wild type PTK6 and MYC-tagged PTK6 SH2 domain mutants and a plasma membrane marker tdTomato PMV-SeQ [19] were transiently expressed in LNCaP, DU-145, PC3, and BPH1 cells and localization was assessed by immunofluorescence (Fig. 2A). Cells were stained for transfected PTK6 protein using the MYC-tag antibody. Pearson Correlation Coefficients (PCC) were determined to quantify colocalization MYC-tagged PTK6 and tdTomato PMV-SeQ, and are presented for each construct in each cell line (Fig. 2A, lower panel). Fluorescence intensity profiles (Fig. S3) were extracted from the position shown by the red line on the cell in the corresponding image. Wild type PTK6 and PTK6 R131A/R136A and PTK6 R132A have similar patterns of intracellular localization and are found in the cytoplasm and at membranes. While PTK6 H126A was expressed at low levels, predominantly in the cytoplasm, R132A and R131A/R136A mutants showed increased membrane fluorescent intensity compared with wild type PTK6.

To further evaluate the impact of the SH2 domain mutations on PTK6 intracellular localization, transfected LNCaP cells were fractionated into cytoplasmic (CP), membrane (M), nuclear (N) and cytoskeletal (CS) fractions, and these were probed for expression of the PTK6 constructs, and control proteins for the cellular fractionation, including the Na^ +^ /K^+^ ATPase (plasma membrane), α-tubulin (cytoplasm), and histone H3 (nucleus). Wild type PTK6, PTK6 R131A/R136A, and PTK6 R132A were detected in the cytoplasmic and membrane fractions, with a higher fraction of the PTK6 R132A and R131A/R136A mutants appearing in the membrane compartment (Fig. 2B, C). In contrast, a reduced fraction of the H126A mutant was present in the cytoplasmic fraction with no detectable expression at the membrane, consistent with the altered membrane-associated immunofluorescence detected by confocal microscopy (Fig. 2A). Decreased membrane localization of the PTK6 H126A PY binding mutant compared with the wild type protein (Fig. 2B, C) may be explained in part by reduced SH2 domain mediated protein-protein interactions at the plasma membrane [25]. Mutation of the previously identified lipid binding residues in PTK6 does not impair plasma membrane localization in cells, while disruption of PTK6 phosphotyrosine binding leads to reduced membrane localization.

### The PTK6 SH2 domain is critical for protein turnover

**Fig. 3:**
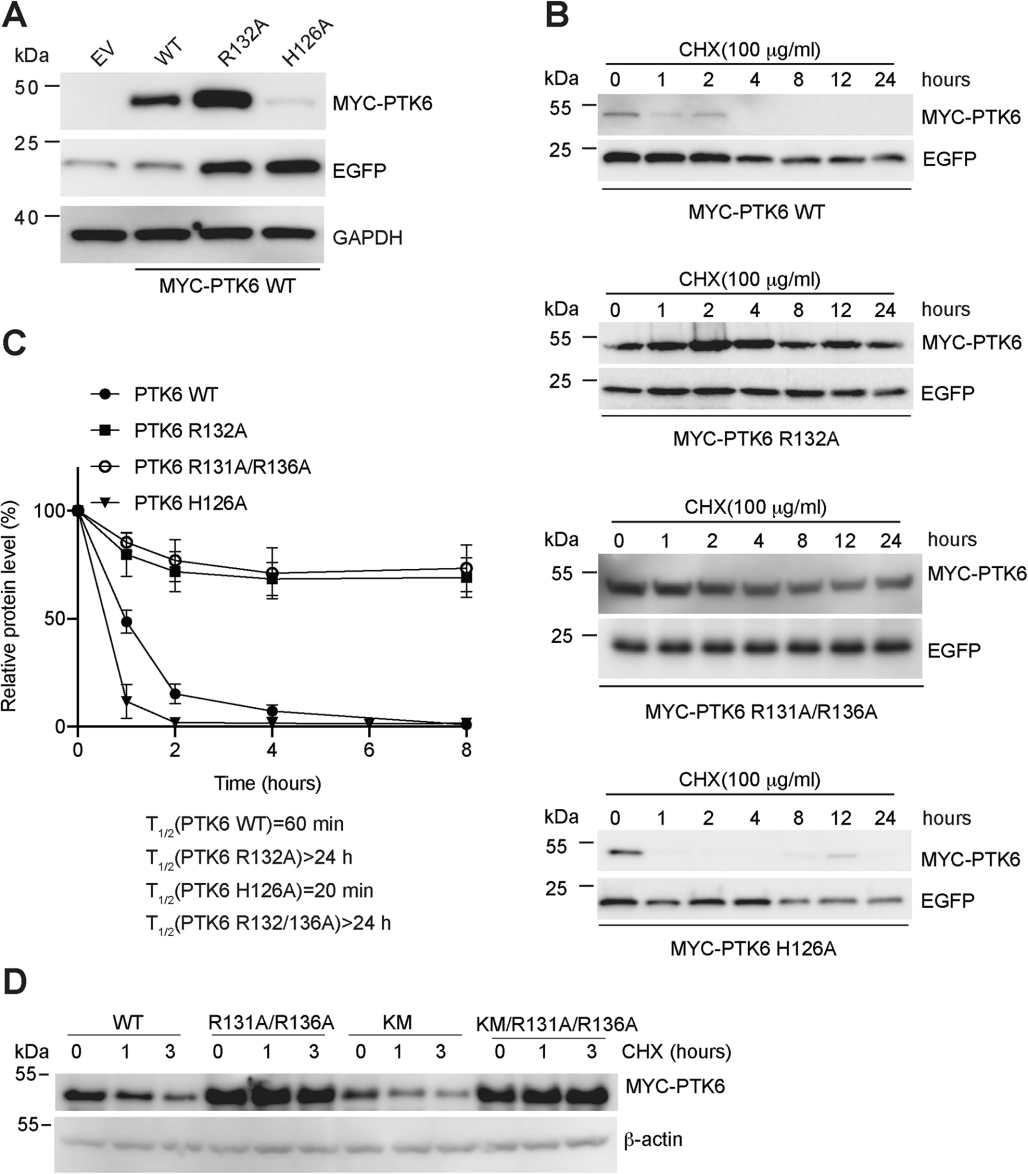
PTK6 R131A/R136A and PTK6 H126A exhibit altered stability compared with wild type PTK6. **A** LNCaP cells were cotransfected with constructs expressing EGFP, empty vector, and wild type or mutant (R132A or H126A) MYC-tagged PTK6. MYC-PTK6 and EGFP levels were analyzed by immunoblotting. EGFP served as a transfection control and GAPDH as a loading control. Reduced H126A levels were not due to poor transfection. **B** LNCaP cells transiently expressing MYC-PTK6 WT or MYC-PTK6 mutants were treated with 100 μM cycloheximide at 24 h post transfection. Cells were lysed at indicated times and MYC-PTK6 levels were analyzed by immunoblotting. **C** The half-lives of PTK6 mutants were calculated using one phase decay exponential equation in GraphPad Prism normalized to EGFP protein levels. Statistics were calculated by paired two-way ANOVA with multiple comparisons, *N* = 3 independent experiments. **D** LNCaP cells transfected with MYC-PTK6 WT or MYC-PTK6 mutants (R131A/R136A, kinase dead KM or KM/R131A/R136A) were treated with 100 μM cycloheximide, lysed at indicated times, and analyzed by immunoblotting. β-actin served as a loading control.

Immunofluorescence and immunoblotting data suggested that PTK6 H126A is expressed at lower levels than wild type PTK6, while expression of the PTK6 arginine mutants is higher (Fig. 2). To determine if differences in expression could be due to differences in transfection efficiency, LNCaP cells were cotransfected with PTK6 WT, PTK6 R132A, or PTK6 H126A and an EGFP construct as a control for transfection efficiency (Fig. 3A). Although EGFP is highly expressed, PTK6 H126A expression is much lower compared with wild type PTK6, while PTK6 R132A is expressed at higher levels (Fig. 3A).

To examine stability of PTK6 WT, PTK6 R132A, PTK6 H126A, and PTK6 R131A/R136A, transfected LNCaP cells were treated with 100 μM cycloheximide (CHX), a protein synthesis inhibitor, starting at 24 h post transfection when highest levels of PTK6 H126A are detected. Cells were harvested at 0, 1, 2, 4, 8, 12 and 24 h after the start of CHX treatment and levels of ectopically expressed proteins were analyzed by immunoblotting (Fig. 3B). A construct encoding EGFP was transfected as a control and MYC-PTK6 levels were normalized relative to EGFP expression. The half-lives of PTK6 WT, PTK6 R131A/R136A, and PTK6 H126A were calculated by non-linear regression (one phase decay) in GraphPad Prism (Fig. 3C). Our results show that the PTK6 R131A/R136A and R132A mutants have a much longer half-life than wild type PTK6, whereas PTK6 H126A has a shorter half-life than the wild type protein. The half-life of PTK6 WT is 1 h, which agrees with a previous report [26]. The half-life of PTK6 R131A/R136A and R132A is longer than 24 h, which suggests that turn-over of these mutants is significantly reduced. PTK6 H126A is degraded faster than the wild type protein with a half-life of only 20 min. To determine if PTK6 activity contributes to its stability, we compared protein levels of PTK6 WT and PTK6 KM with and without the arginine mutations in cells treated with CHX. Both wild type and kinase-dead PTK6 with arginine mutations exhibit increased stability (Fig. 3D).

### PTK6 protein levels are regulated by Cullin-1-RING ligase mediated ubiquitin-proteasome degradation

**Fig. 4:**
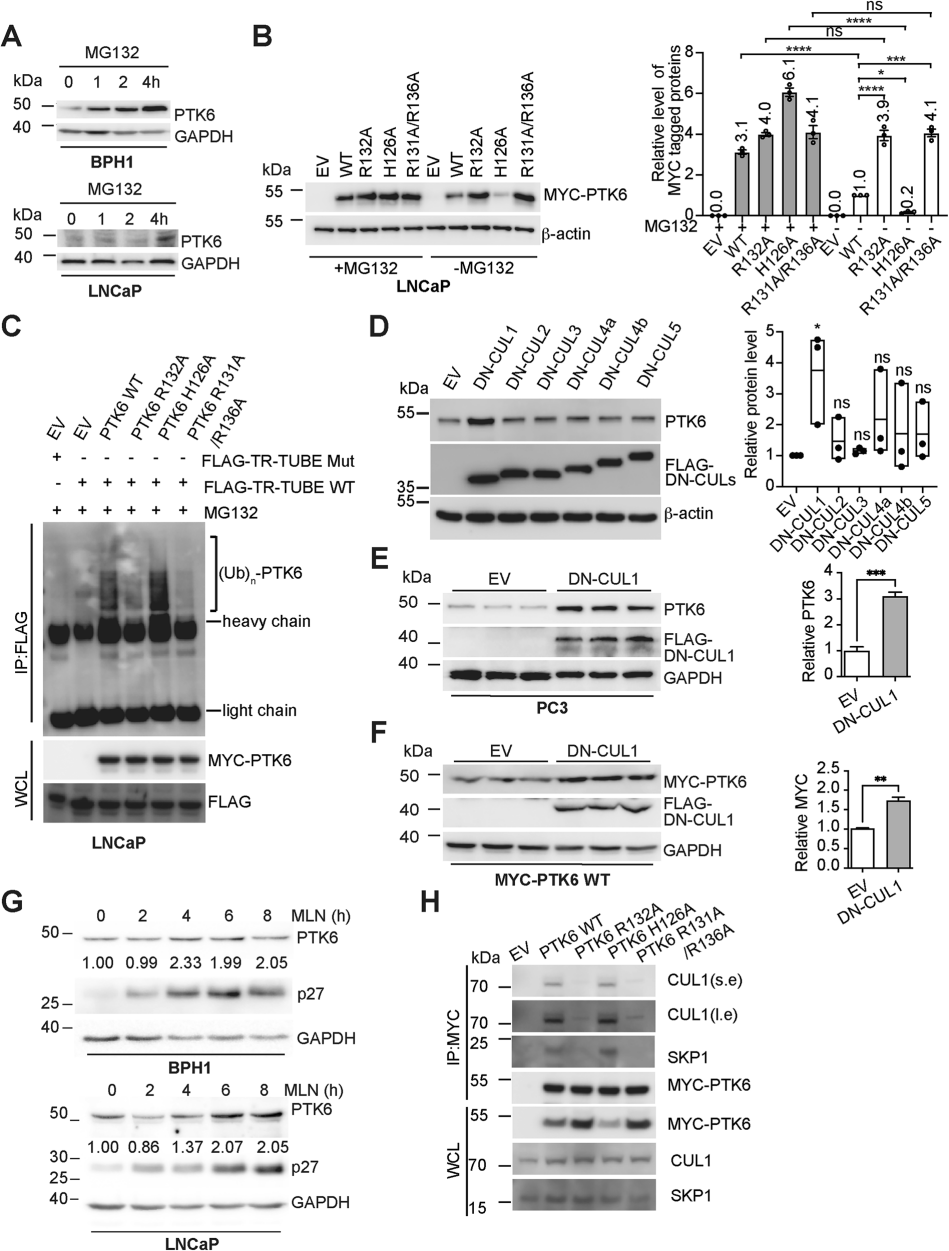
Arginine residues in the PTK6 SH2 domain regulate its proteasomal degradation and association with a Cullin-1-Ring Ubiquitin Ligase Complex. **A** BPH1 and LNCaP cells were treated with 10 μM MG-132 and endogenous PTK6 protein levels were analyzed by immunoblotting at the indicated times. **B** LNCaP cells transfected with empty vector (EV), MYC-PTK6 WT and MYC-PTK6 mutants were treated with MG-132 for 6 h. Lysates were immunoblotted with MYC-tag antibody (left panel). Protein levels were calculated after normalization to β-actin, with the level of untreated WT PTK6 (without MG-132) set to 1.0 (right panel). Statistics were calculated by one-way ANOVA followed by Tukey’s test, *N* = 3 independent experiments. **C** LNCaP cells co-transfected with wild type or mutant MYC-tagged PTK6 constructs and wild type FLAG-tagged or mutant TR-TUBE, were treated for 4 h with 10 μM MG-132. FLAG-tagged TR-TUBE coupled ubiquitin complexes were immunoprecipitated and blotted with PTK6 antibody. **D** Endogenous PTK6 protein levels were examined in LNCaP cells expressing FLAG-tagged dominant negative (DN) human Cullins 1–5. Protein levels were calculated after normalization to β-actin, with the level of PTK6 in the EV control transfection set to 1.0 (right panel) in three independent experiments. Statistics were calculated by one-way ANOVA followed by Tukey’s test. **E** Lysates from PC3 cells transfected with EV or a construct expressing FLAG-tagged dominant negative Cullin-1 (DN-CUL1) were immunoblotted for endogenous PTK6. Protein levels were calculated after normalization to GAPDH, with the level of EV set to 1.0 (right panel). Statistics were calculated by Student’s t test, *N* = 3 independent experiments. **F** HEK293 cells cotransfected with MYC-PTK6 WT, and empty vector or FLAG-DN-CUL1 were blotted for MYC-PTK6. Each lane represents an independent transfection, and MYC-PTK6 levels were normalized to GAPDH. Statistics were calculated by Student’s t test. **G** LNCaP and BPH1 prostate cells were treated with MLN4924 (MLN) to inhibit Cullin-RING ligases for the indicated times, and endogenous PTK6 protein levels were examined. p27 served as a positive control. Protein levels were normalized to β-actin, with the level of untreated wild-type (WT) PTK6 at the 0-hour timepoint set to 1.0. **H** Lysates from LNCaP cells transfected with MYC-tagged PTK6 WT or mutant constructs were subjected to immunoprecipitation with MYC-tag antibody and blotted for CUL1 and SKP1 at 24 h post transfection; s.e. (short exposure), l.e. (long exposure).

The ubiquitin-proteasome system (UPS) was previously reported to regulate wild type PTK6 [26]. To determine the impact of inhibiting the proteasome in prostate cells expressing endogenous PTK6, we treated LNCaP cells, a prostate cancer cell line with low levels of endogenous PTK6, and BPH1 cells, a benign prostate hyperplasia cell line that expresses higher levels of PTK6, with MG-132. Cells were lysed 0, 1, 2, and 4 h after the beginning of treatment, and PTK6 protein levels were analyzed by immunoblotting. We observed an increase in endogenous PTK6 levels in a time dependent manner in both prostate cell lines (Fig. 4A).

Since the PTK6 phosphotyrosine-binding mutant PTK6 H126A has a very short half-life, and the R131A/R136A and R132A mutants have long half-lives (Fig. 3), we asked if PTK6 mutants are differentially regulated by the cellular proteasome machinery (Fig. 4B). LNCaP cells were transfected with WT, R131A/R136A, R132A and H126A PTK6 expression constructs and treated with 10 μM MG-132 post transfection. Total cell lysates were prepared at 24 h after MG132 addition and subjected to immunoblotting. Inhibition of proteasomal degradation led to increased expression of wild type MYC-PTK6, consistent with the endogenous protein, and a dramatic increase in PTK6 H126A protein levels (Fig. 4B).

To determine if PTK6 is a substrate for polyubiquitination, we used the Trypsin Resistant (TR)-Tandem Ubiquitin-Binding Entities (TUBE) system [27]. LNCaP cells were cotransfected with the MYC-tagged PTK6 constructs and FLAG-tagged TR-TUBE WT or a ubiquitin binding-deficient TR-TUBE mutant. TR-TUBE WT enriches selectively for polyubiquitinated proteins of linkage type K48, and to a lesser extent K63, or other ubiquitin-chain types, whereas TR-TUBE mutant fails to do so, allowing sensitive detection of ubiquitinated PTK6 species. PTK6 H126A and to a lesser extent wild type PTK6 exhibited high levels of polyubiquitination, while PTK6 R132A and R131A/R136A showed polyubiquitination levels comparable to the empty vector control (Fig. 4C). Consistent with this, similar levels of K48-linked polyubiquitinated proteins were enriched by TR-TUBE but the TR-TUBE mutant failed to enrich polyubiquitinated proteins (Suppl. Fig. S4A). This result is expected, as TR-TUBE captures all polyubiquitinated proteins in addition to PTK6 in mammalian cell-based assays, indicating that differences observed in PTK6 polyubiquitination when probed for PTK6 reflect selective enrichment of polyubiquitinated PTK6.

To determine if a Cullin-RING E3 ligase (CRL) regulates PTK6, we expressed dominant negative (DN-) Cullins 1–5 in LNCaP cells. Only DN-Cullin 1 significantly increased PTK6 protein levels (Fig. 4D), a result confirmed with endogenous (Fig. 4E) and transfected (Fig. 4F) PTK6 in PC3 cells (Fig. 4E). Furthermore, treatment of LNCaP and BPH1 cells with the Cullin-1-RING ligase (CRL1) inhibitor MLN4924 caused a time-dependent increase in PTK6 protein expression (Fig. 4G) without affecting mRNA levels (Fig. S4B), mimicking proteasomal inhibition with MG132 (Fig. 4A). These data underscore contributions of a CRL1 complex in regulation of PTK6 protein stability.

We examined PTK6 association with proteins in the CRL1 complex. LNCaP cells were transfected with PTK6 WT, R132A, R131A/R136A, or H126A expression constructs, or empty vector and lysed at 24 h post transfection. PTK6 was immunoprecipitated using MYC-tag antibodies and association of SKP1 and CUL1 with PTK6 was examined by immunoblotting (Fig. 4H). Our results show both PTK6 WT and PTK6 H126A co-immunoprecipitate with the CRL1 components SKP1 and Cullin-1, consistent with shorter half- lives (Fig. 3), and their ability to undergo polyubiquitination (Fig. 4C). Furthermore, Cullin-1 and SKP1 were undetectable in immunocomplexes containing PTK6 R131A/R136A or PTK6 R132A consistent with increased stability (Fig. 3) and undetectable polyubiquitination (Fig. 4C).

### Arginine residues 131, 132 and 136 in the PTK6 SH2 domain are methylated

**Fig. 5:**
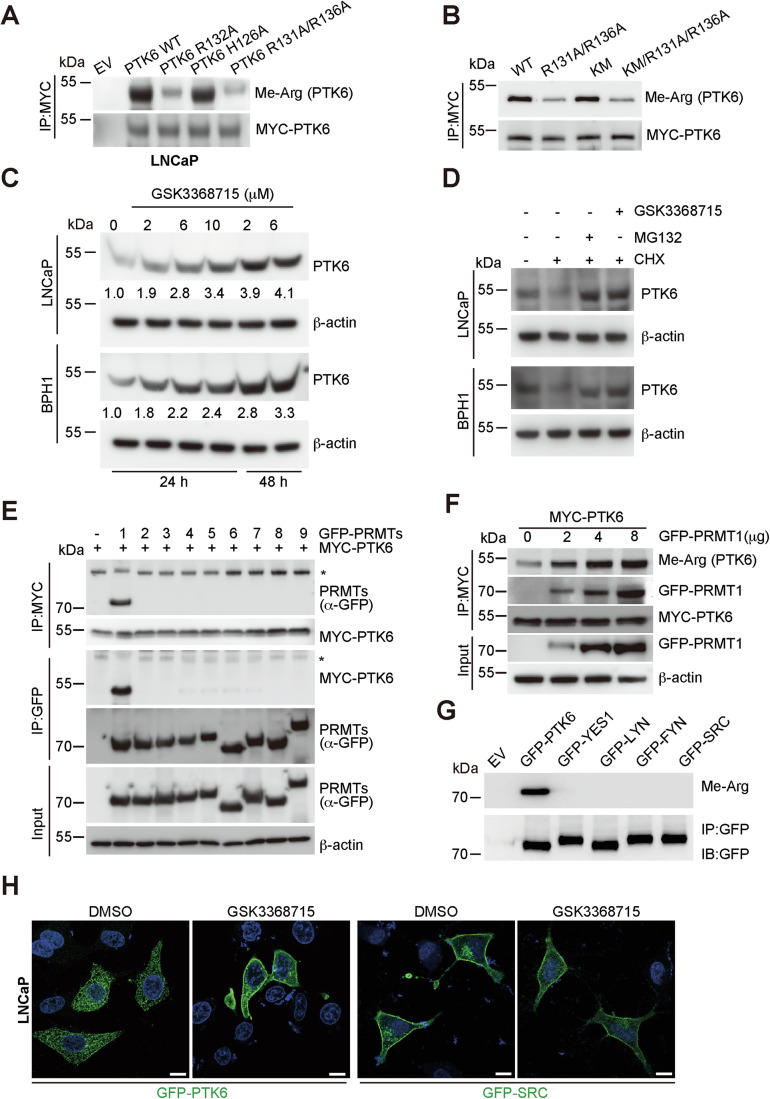
PRMT1 methylates PTK6 arginine residues and regulates its stability and intracellular localization. **A**, **B** LNCaP cells transfected with EV, WT or mutant MYC-PTK6 expression constructs were lysed at 24 h post transfection. MYC-tagged PTK6 proteins were immunoprecipitated with MYC-tag antibody and immunoblotted with methyl arginine (Me-Arg) antibody. **C** LNCaP and BPH1 cells were treated with indicated doses of GSK3368715 and lysed at 24 or 48 h. Immunoblotting was done to examine endogenous PTK6 protein. Fold change in PTK6 relative to β-actin is indicated. **D** LNCaP and BPH1 cells were treated with 100 μg/ml cycloheximide, ± 10 μM MG-132 or 6 μM GSK3368715 and lysed at 24 h. In the absence of protein synthesis, MG-132 or GSK3368715 treatment leads to increased endogenous PTK6 protein. **E** Reciprocal immunoprecipitations (MYC-tag or GFP) were performed with lysates from LNCaP cells cotransfected with wild type MYC-PTK6 and GFP-tagged PRMTs 1–9, and immunoblotting was done with indicated antibodies. Asteriks denote nonspecific bands. **F** Immunoprecipitations using the MYC-tag antibody and lysates from LNCaP cells transfected with wild type MYC-PTK6 and increasing amounts of the GFP-PRMT1 expression construct were subjected to immunoblotting as indicated. GFP-PRMT1 coimmunoprecipitates with MYC-PTK6, and increased levels of PRMT1 expression correspond with increased PTK6 methylation on arginine residues. **G** Lysates from LNCaP cells transfected with GFP-tagged PTK6, YES, LYN, FYN or SRC kinase expression constructs were subjected to immunoprecipitation with GFP antibody and immunoblotted with methyl arginine antibody. Only GFP-PTK6 exhibits methylation on arginine residues. **H** LNCaP cells were transfected with GFP-PTK6 or GFP-SRC for one hour and then incubated with DMSO or 6 μM GSK3368715. Live cell imaging was performed at 24 h post inhibitor treatment to visualize GFP-tagged proteins. Scale bar: 10 μm.

Since PRMTs may target sequences rich in glycine residues, which are found between PTK6 R132 and R136, we probed wild type and mutant PTK6 for arginine methylation. LNCaP cells were transfected with empty vector, and MYC-tagged PTK6 expression constructs (WT, R132A, H126A, and R131A/R136A), and immunoblotting of immunoprecipitated with an anti-methyl-Arg antibody revealed strong arginine methylation on wild type and H126A PTK6, but not on R132A or R131A/R136A (Fig. 5A). Furthermore, kinase dead PTK6 KM exhibited methylation levels comparable to wild type, indicating PTK6 activity does not impact its methylation (Fig. 5B).

To determine if arginine methylation impacts endogenous PTK6 expression, LNCaP and BPH1 cells were treated with the type I PRMT inhibitor GSK3368715 for 24 or 48 h. Inhibition of type-1 methyltransferases increased protein levels but not mRNA levels (Fig. 5C, S4B). GSK3368715 and the proteasome inhibitor MG132 similarly stabilized PTK6 in the presence of cycloheximide (Fig. 5D). These data suggest that PTK6 SH2 domain arginine methylation is required to recruit a CRL1 complex; consequently, inhibition of methylation or mutation of the 131, 132 and 136 arginine residues is sufficient to promote PTK6 protein stability (Fig. 3C and Fig. 5C, D).

There are nine different PRMTs, with six type-1 enzymes (PRMT1, 2, 3, 4, 6, and 8) that catalyze formation of asymmetric dimethyl arginine [28]. To identify which PRMT(s) target PTK6, MYC-tagged PTK6 was coexpressed with GFP-tagged PRMTs 1–9. Coimmunoprecipitations revealed that only PRMT1 associated with PTK6 (Fig. 5E). Furthermore, increasing GFP-PRMT1 expression led to a dose dependent increase in MYC-PTK6 arginine methylation, supporting our finding that PRMT1 targets PTK6 (Fig. 5F).

To determine if arginine methylation is specific for PTK6, or a general occurrence in related intracellular tyrosine kinases, we transfected LNCaP cells with GFP-tagged PTK6 or GFP-tagged SRC family kinases YES, LYN, FYN, and SRC. GFP-tagged proteins were immunoprecipitated and subjected to immunoblotting with the methyl-arginine antibody. Only PTK6 exhibited methylation on arginine residues (Fig. 5G). SRC contains conserved arginine residues in its SH2 domain (158-**R-R-**E-S-E-**R**-163) but they are not flanked by glycine residues as in PTK6 (131-**R-R**-A-G-G-**R**-136).

We next asked if arginine methylation influences PTK6 intracellular localization. LNCaP cells were transfected with either GFP-PTK6 or GFP-SRC constructs and treated with DMSO or the arginine methylation inhibitor GSK3368715 for one hour, and GFP-tagged proteins were visualized. Interestingly, inhibition of arginine methylation promoted accumulation of PTK6 at the plasma membrane but had little impact on SRC intracellular localization (Fig. 5H).

### Methylation of arginine residues in the PTK6 SH2 domain is important for its association with substrates and signaling

**Fig. 6:**
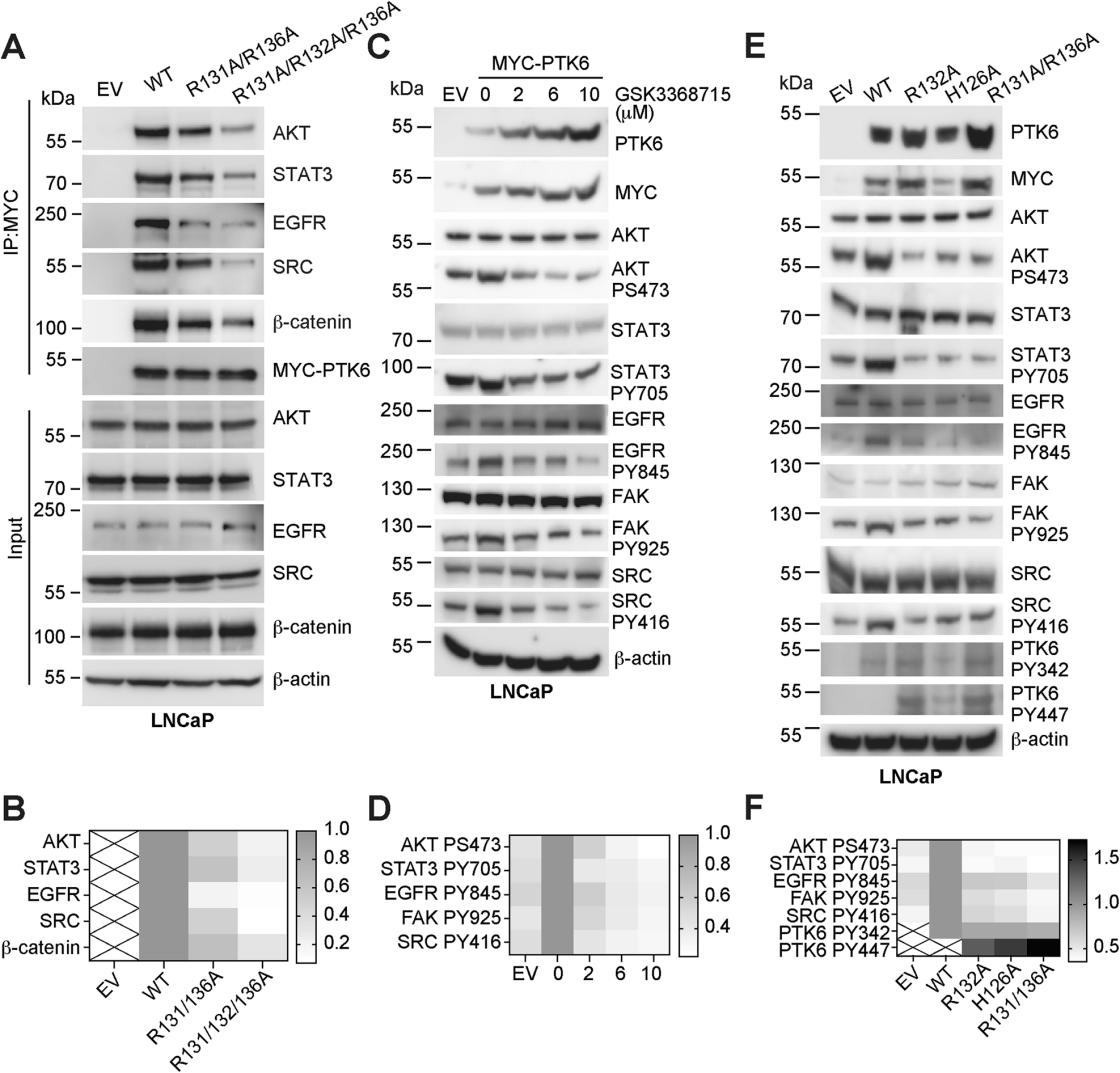
Arginine methylation positively regulates PTK6 substrate association and downstream activity. **A**, **B** LNCaP cells expressing MYC-tagged WT and mutant PTK6 constructs were lysed at 24 h post transfection and immunoprecipitations were performed with the MYC-tag antibody followed by immunoblotting with antibodies against known PTK6 substrates to examine PTK6-substrate association. **C**, **D** LNCaP cells treated with increasing doses of the PRMT inhibitor GSK3368715 were lysed at 24 h post treatment. Immunoblotting was performed with indicated antibodies to examine total protein and site specific phosphorylation of known PTK6 substrates. **E**, **F** LNCaP cells were transfected with MYC-tagged WT and mutant PTK6 constructs and lysed at 24 h post transfection. Whole cell lysates were subjected to immunoblotting with indicated antibodies, including phospho-specific antibodies, to examine phosphorylation of PTK6 substrates. **B**, **D**, **F** Normalized intensity levels are shown; (**B**) Coimmunoprecipitated protein/total protein. **D**, **F** Protein phosphorylation levels/total protein levels.

PTK6 phosphorylates substrates like AKT [22], STAT3 (Y705) [29], EGFR (Y845) [30, 31], FAK (Y925) [9] and SRC (Y416) [23]. To examine the importance of the SH2 domain arginine residues on association of PTK6 with its substrates, we expressed MYC-tagged PTK6 WT, R131A/R136 A, and R131A/R132A/R136A in LNCaP cells and immunoprecipitated with anti-MYC-tag to examine coimmunoprecipitation of PTK6 substrates by immunoblotting (Fig. 6A). Mutation of arginine residues targeted by PRMT1 led to decreased substrate binding, indicating that these arginine residues play an important role in PTK6 substrate association. The amount of substrate that coimmunoprecipitated relative to total protein is summarized in the heatmap (Fig. 6B).

To address the impact of arginine methylation on PTK6 activity, LNCaP cells were transfected with empty vector or MYC-tagged wild type PTK6 and treated with the PRMT inhibitor GSK3368715. While inhibition of methylation increased total PTK6 protein levels, it decreased PTK6 substrate phosphorylation and AKT activation [22] (Fig. 6C). Site specific phosphotyrosine (PY) antibodies are available for most PTK6 substrates, except AKT. Phosphorylation of AKT on tyrosine residues results in increased AKT activation, which is measured by examining increased activating phosphorylation on serine 473. The amount of phosphorylated protein relative to total protein is summarized in the heatmap (Fig. 6D). These data support a positive role for arginine methylation in regulation of PTK6 substrate phosphorylation.

In LNCaP cells, wild type PTK6 increases site-specific phosphorylation (PY) of its substrates, while expression of the SH2 domain mutants (H126A, R131A/136 A, R132A) results in lower substrate phosphorylation and less AKT activation. (Fig. 6E). A heat map below the blots summarizes levels of phosphorylation (Fig. 6F). Using antibodies against PTK6 activation (PY342) and autoinhibitory (PY447) phosphorylation sites, increased inhibitory phosphorylation was detected for PTK6 R131A/R136A, and R132A, which may contribute to decreased kinase activity.

**Fig. 7:**
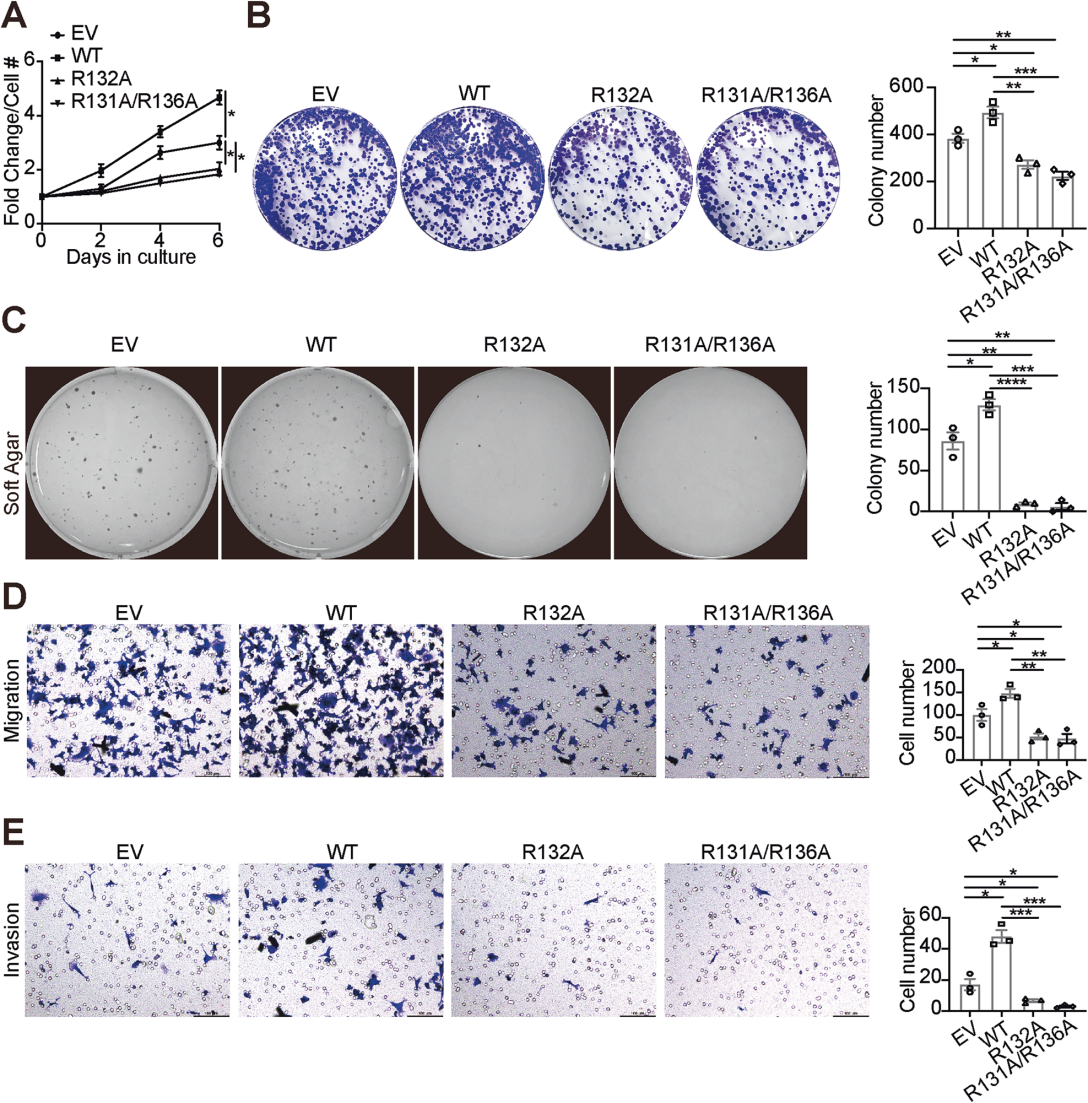
PTK6 SH2 domain arginine residues regulate its oncogenic activities in prostate cancer cells. LNCaP cells stably expressing wild type or mutant PTK6 were generated. Cells were counted at the indicated times, and the fold change in cell number is shown (**A**). Mutation of PTK6 R132 or R131/R136 reduces PTK6 regulated LNCaP cell colony formation (**B**), anchorage independent growth in soft agar (**C**), transwell migration (**D**) and transwell invasion (**E**). Representative images are shown for three independent experiments. For (**B**–**E**), quantification is shown as bar graphs from three replicates (right panels). Statistics were calculated by paired two-way ANOVA with multiple comparisons (**A**), or one-way ANOVA followed by Tukey’s test (**B**–**E**). Error bars: SEM.

While mutation of PTK6 R132 or R131/R136 or treatment with the PRMT inhibitor GSK3368715 leads to increased levels of PTK6 protein, methylation deficient PTK6 has diminished ability to engage its substrates (Fig. 6). To begin to address the biological significance of PTK6 arginine methylation, we examined the transforming potential of the PTK6 arginine methylation mutants in LNCaP cells. Using selected stable cell pools, we detect reduced proliferation (Fig. 7A), colony formation (Fig. 7B), anchorage independent growth (Fig. 7C), migration (Fig. 7D), and invasion (Fig. 7E) when PTK6 arginine mutants (R132A, R131A/R136A) are expressed, compared with wild type PTK6. These data suggest that arginine methylation is critical for PTK6-mediated transformation.

## Discussion

PTK6 has context-specific oncogenic or tumor-suppressive roles depending on its intracellular localization. Several studies suggest that targeting PTK6 in breast and prostate cancer may have therapeutic benefits (reviewed in [1, 32, 33]). Membrane-associated active PTK6 is present in breast tumors but not normal breast tissue [12], and disruption of *Ptk6* impairs tumorigenesis in a mouse breast cancer model [34]. In prostate cancer cells, membrane association of active PTK6 promotes an epithelial mesenchymal transition and tumor progression [2, 10], while nuclear PTK6 restrains prostate cancer cell growth [5]. Disruption of *Ptk6* in a mouse model of prostate cancer, or inhibition of PTK6 activity in a xenograft model reduced tumor burden [11, 31]. Determining how PTK6 activity is regulated in a spatiotemporal manner is critical for understanding its pro- and anti-oncogenic functions.

The finding that SH2 domains can bind plasma membrane lipids raised the possibility that PTK6 membrane localization might be facilitated by its SH2 domain [14]. We investigated contributions of PTK6 SH2 domain amino acid residues H126 and R131/R136 and R132 to its membrane association, either through phosphotyrosine binding or direct membrane-lipid interaction, respectively. While PTK6 amino acid residue H126 mediates binding to phosphotyrosine [15], residues R131 and R136 were identified as important for lipid binding, but with low phosphoinositide selectivity, in an in vitro plasma membrane mimetic vesicle assay [14]. Mutations of PTK6 R132 or R131/R136 did not alter PTK6 lipid binding in lipid overlay assays (Fig. 1 and S1) or PTK6 localization to the plasma membrane or subcellular cell fractions in prostate cancer cells (Fig. 2). However, mutation of H126 reduced PTK6 membrane localization (Fig. 2C), suggesting importance of protein-protein interactions for membrane localization.

Mutations at R131 and R132/R136 dramatically increase PTK6 protein stability (Fig. 3). We show PTK6 is regulated by the proteasome (Fig. 4A, B) and the SH2 arginine residues are important for ubiquitination (Fig. 4C), and association with a CUL1/SKP1 E3-ligase complex (Fig. 4H). In contrast, mutation of amino acid residue H126 decreases PTK6 protein half-life, potentially exposing an E3-ligase binding site. Identifying the specific CRL1 components may enable development of tissue specific PROTACs to target oncogenic PTK6 in cancers.

Protein arginine methylation is an important regulator of transcription, RNA metabolism and signaling [reviewed in [16, 17, 28]]. It can positively and negatively regulate protein-protein interactions [35]. Inhibiting arginine methylation or the proteasome led to a similar increase in endogenous PTK6 expression in prostate cell lines (Fig. 5D). We determined that arginine residues within the PTK6 SH2 domain are targets of PRMT1 (Fig. 5E, F), and our findings suggest that methylated R131, R132, and R136 provide structural or electrostatic cues to recruit the ubiquitin proteasome system. Many signaling kinases undergo rapid ubiquitin-dependent turnover following activation as a mechanism to limit signaling duration [36, 37]. Consistent with this paradigm, increased degradation of methylated PTK6 may reflect enhanced turnover of the activated PTK6 pool rather than a direct destabilizing effect of methylation itself. Although the E3 ligase responsible for PTK6 ubiquitination has not yet been defined in this context, our data support a model in which arginine methylation promotes PTK6 activation, which is subsequently coupled to proteasomal degradation.

Arginine methylation has been shown to regulate activities of some receptor tyrosine kinases, including the epidermal growth factor receptor EGFR [38], FLT3-ITD (FMS-like tyrosine kinase 3 with a internal tandem duplication) [39], and vascular endothelial growth factor receptor 2 (VEGFR2) [40]. However, we did not detect arginine methylation in PTK6-related intracellular tyrosine kinases including SRC and the SRC-family members YES, LYN or FYN (Fig. 5G). Inhibition of arginine methylation increased PTK6 but not SRC membrane localization (Fig. 5H); it will be important to determine if this is a byproduct of increased PTK6 stabilization or if other methylation-dependent mechanisms regulate PTK6 intracellular localization.

Although expressed at higher levels, methyl-deficient PTK6 exhibits increased phosphorylation on tyrosine residue 447 in its C-terminal tail, and we detected reduced substrate phosphorylation and binding to substrates (Fig. 6). Like SRC-family kinases, phosphorylation of the PTK6 C-terminal tyrosine is inhibitory and it remains to be determined if arginine methylation helps maintain PTK6 in an active conformation. Arginine methylation positively regulates AKT activity by promoting an open active conformation [41]. In contrast to wild type PTK6, stable expression of PTK6 arginine mutants diminished prostate cancer cell proliferation, anchorage independent growth, migration and invasion (Fig. 7), suggesting that arginine methylation is critical for PTK6 oncogenic activities.

Mutation of amino acid residues H126, R131, R132, and R136 within the PTK6 SH2 domain have been identified in the COSMIC (https://cancer.sanger.ac.uk/cosmic) [42], cBioPortal (http://www.cbioportal.org), [43] and Clinvar (https://www.ncbi.nlm.nih.gov/clinvar/) [44] databases (Table 1). We found that mutation of H126, involved in phosphotyrosine binding, destabilizes PTK6. The H126P mutation, identified in esophageal cancers may destabilize PTK6 to promote tumorigenesis in the esophagus where PTK6 appears to act as a tumor suppressor [45, 46]. Conversely, mutation of PTK6 amino acid residues R131, R132, and R136 found in gastric, lung, mesothelioma, renal clear cell carcinoma, and glioblastoma (Table 1) would be expected to increase PTK6 stability, while also impairing its activity toward substrates. Further research should clarify how PTK6 functions in cancer, through both kinase-dependent and -independent mechanisms. Our findings indicate that the SH2-domain and arginine methylation may drive its context-specific roles, making them promising targets for new therapies.

**Table 1:** Mutations in the PTK6 SH2 domain.

MUTATION	NUMBER	CANCER	DATABASE
H126P	3	Esophageal adenocarcinoma	COSMIC
R131L	1	Gastric adenocarcinoma	COSMIC
R136G	1	Mesothelioma	COSMIC
R131P	1	Lung squamous cell carcinoma	cBioPortal COSMIC
R131Q	1	Clear cell renal cell carcinoma	cBioPortal
R132P	1	Glioblastoma Multiforme	cBioPortal
R136P	Not provided	Not provided	Clinvar
R136W	Not provided	Not provided	Clinvar

PTK6 mutations were identified in the COSMIC [42], cBioPortal [43], and Clinvar [44] databases.

## Supplementary information

Supplementary Text and Figures

Raw data

## Data Availability

The manuscript contains all data described within the text.
