# Supplementary material for: Protein tyrosine kinase-6 is post-translationally regulated by arginine methylation and ubiquitination
Source: Cell Death Dis. 2026 Jun 5;17(1):706. doi: 10.1038/s41419-026-08933-5 (PMC13458159; doi:10.1038/s41419-026-08933-5)

Fig 1B

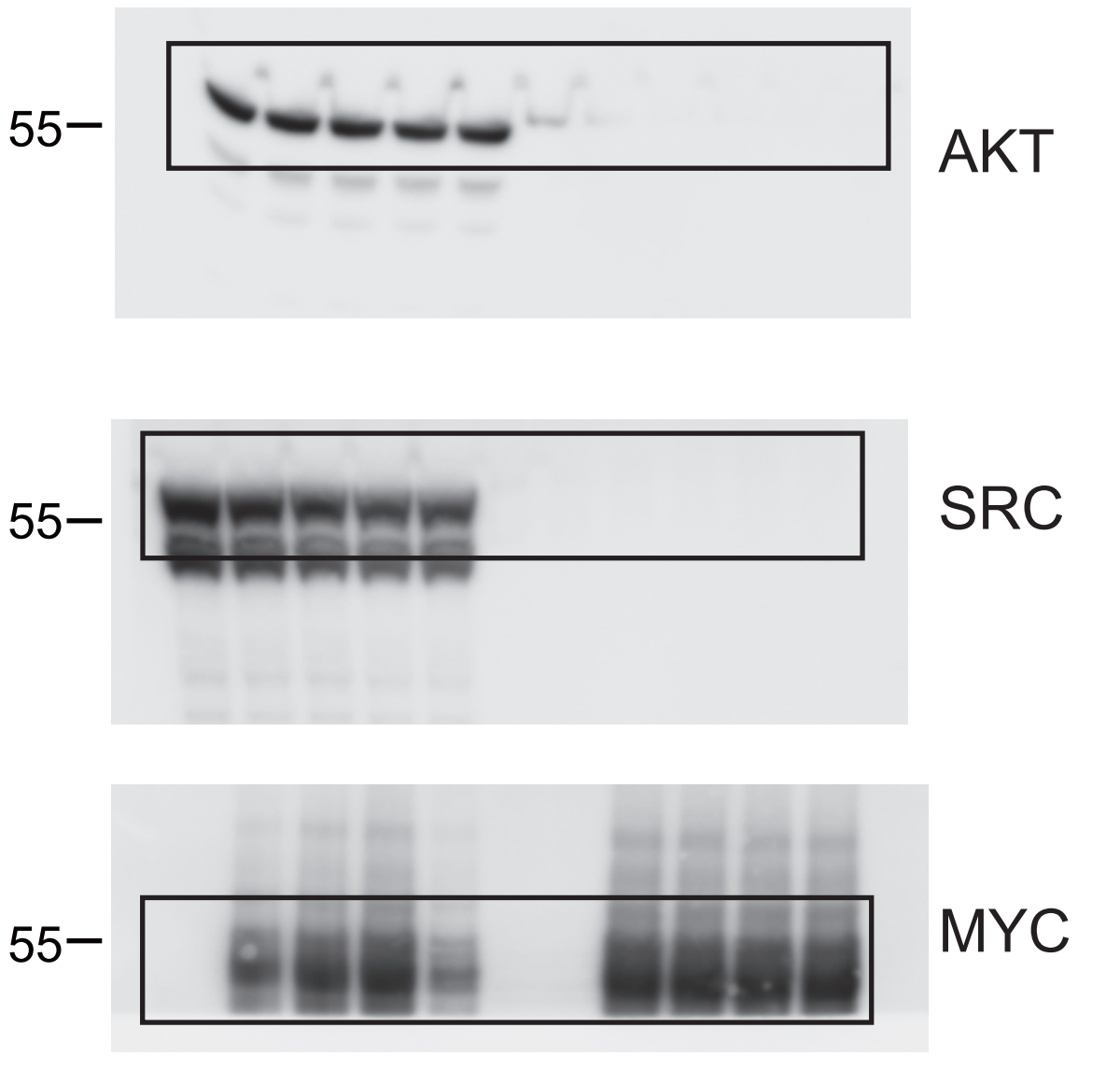

Fig 2B

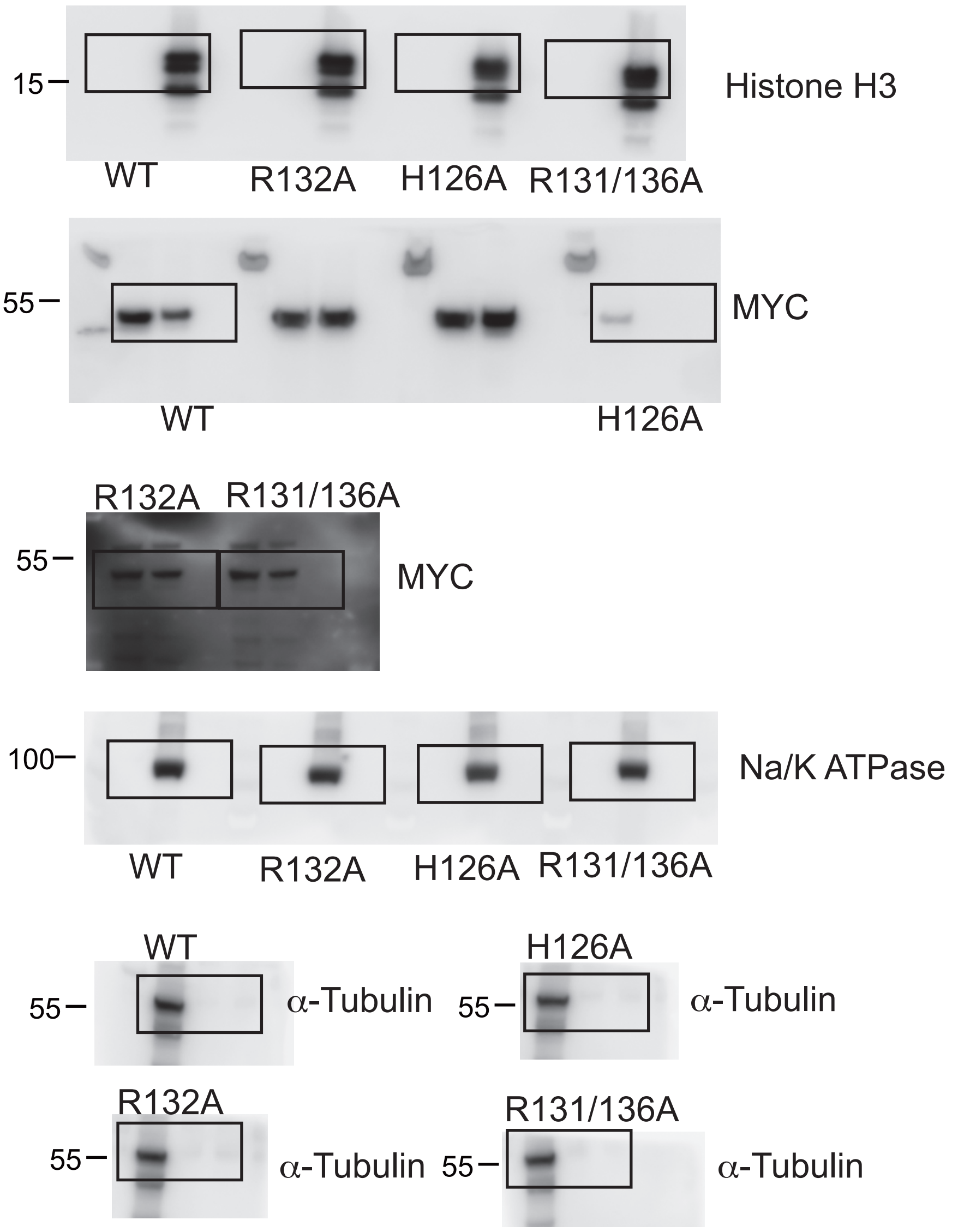

Fig 3A

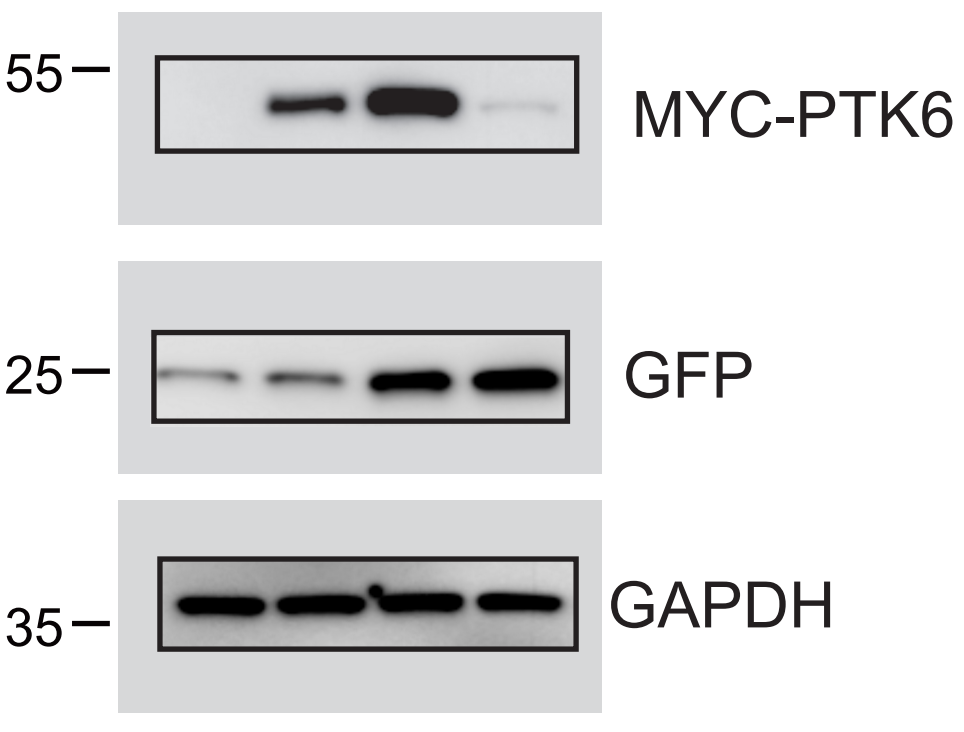

Fig 3B

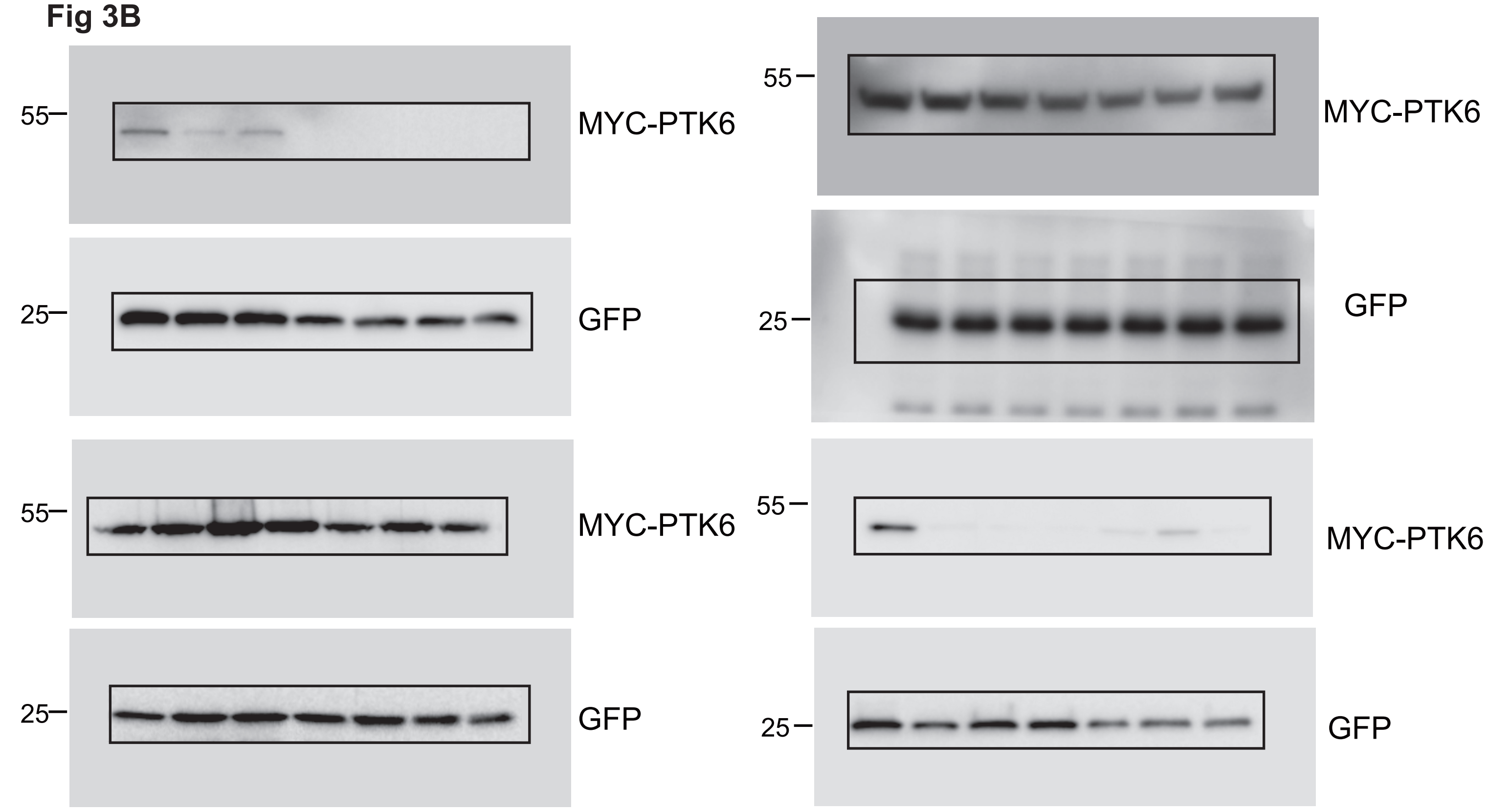

Fig 3C

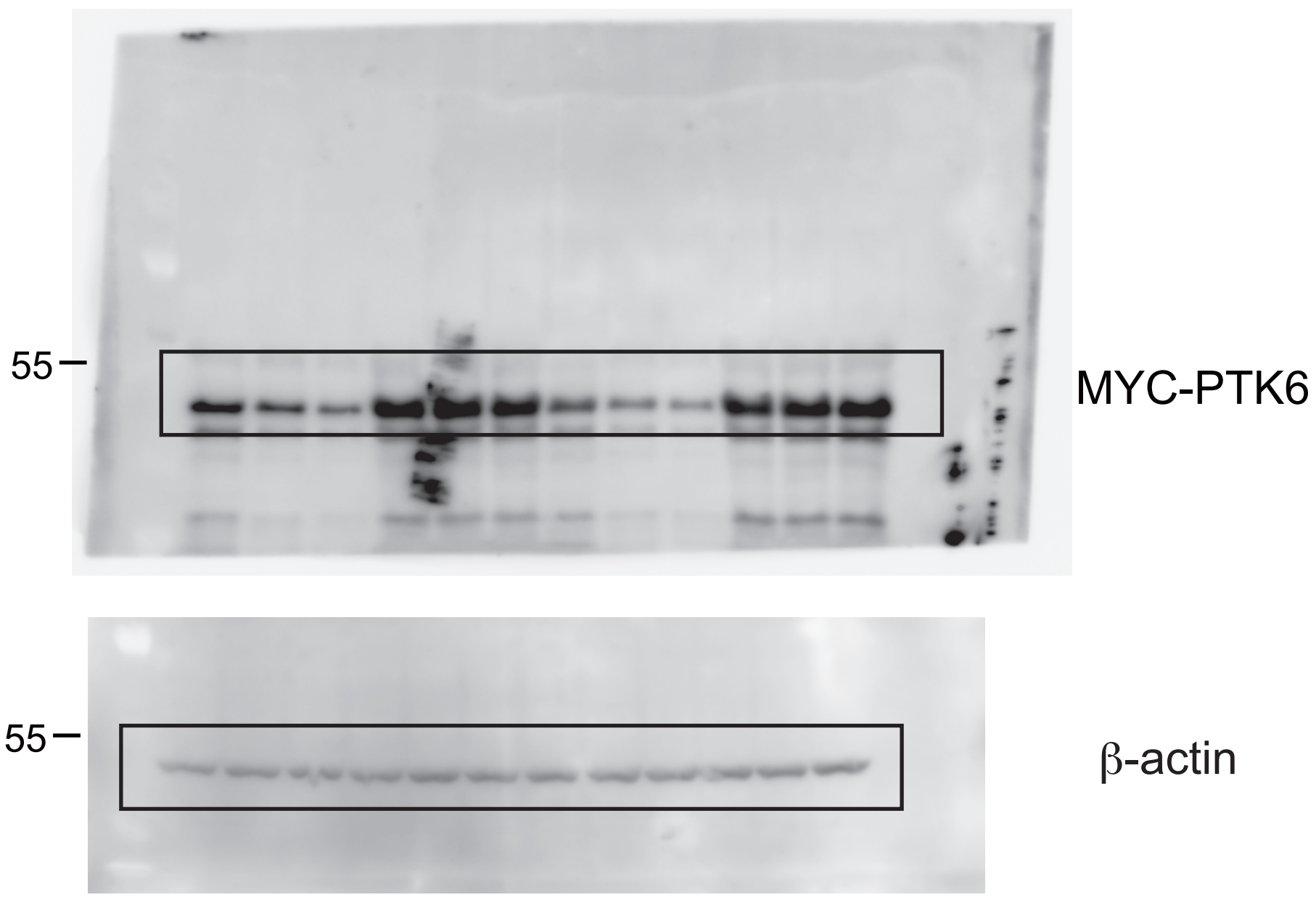

Fig 4A

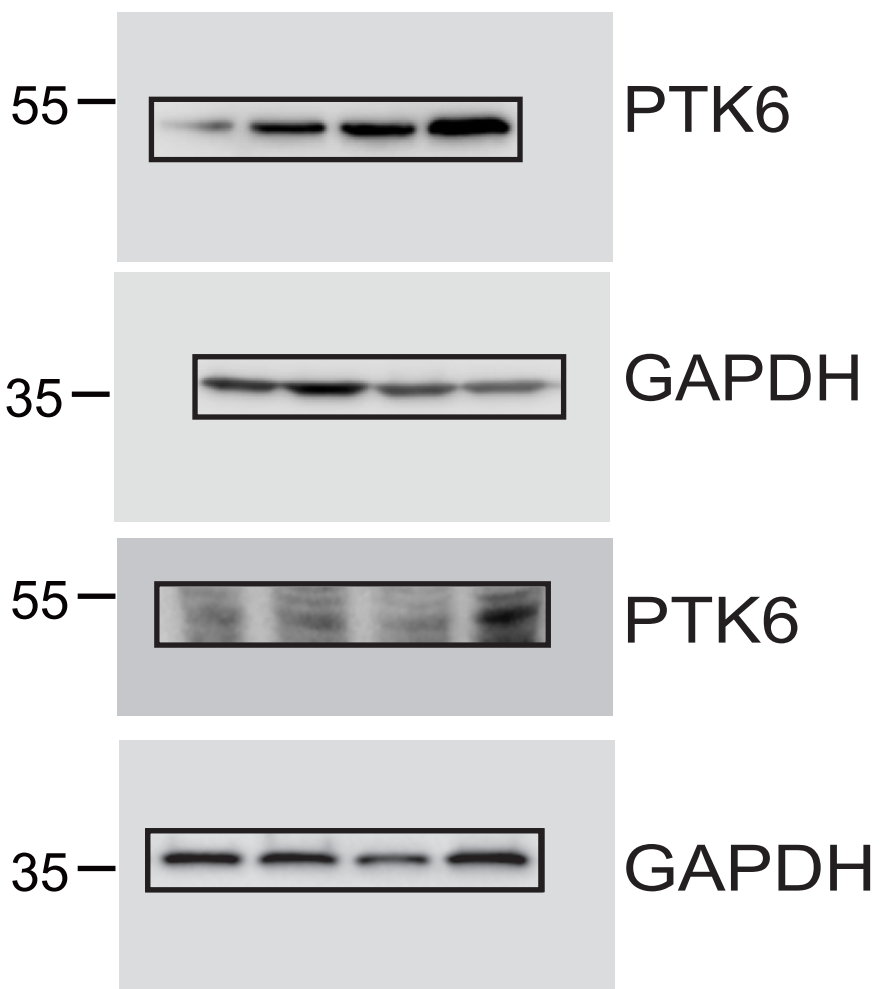

Fig 4B

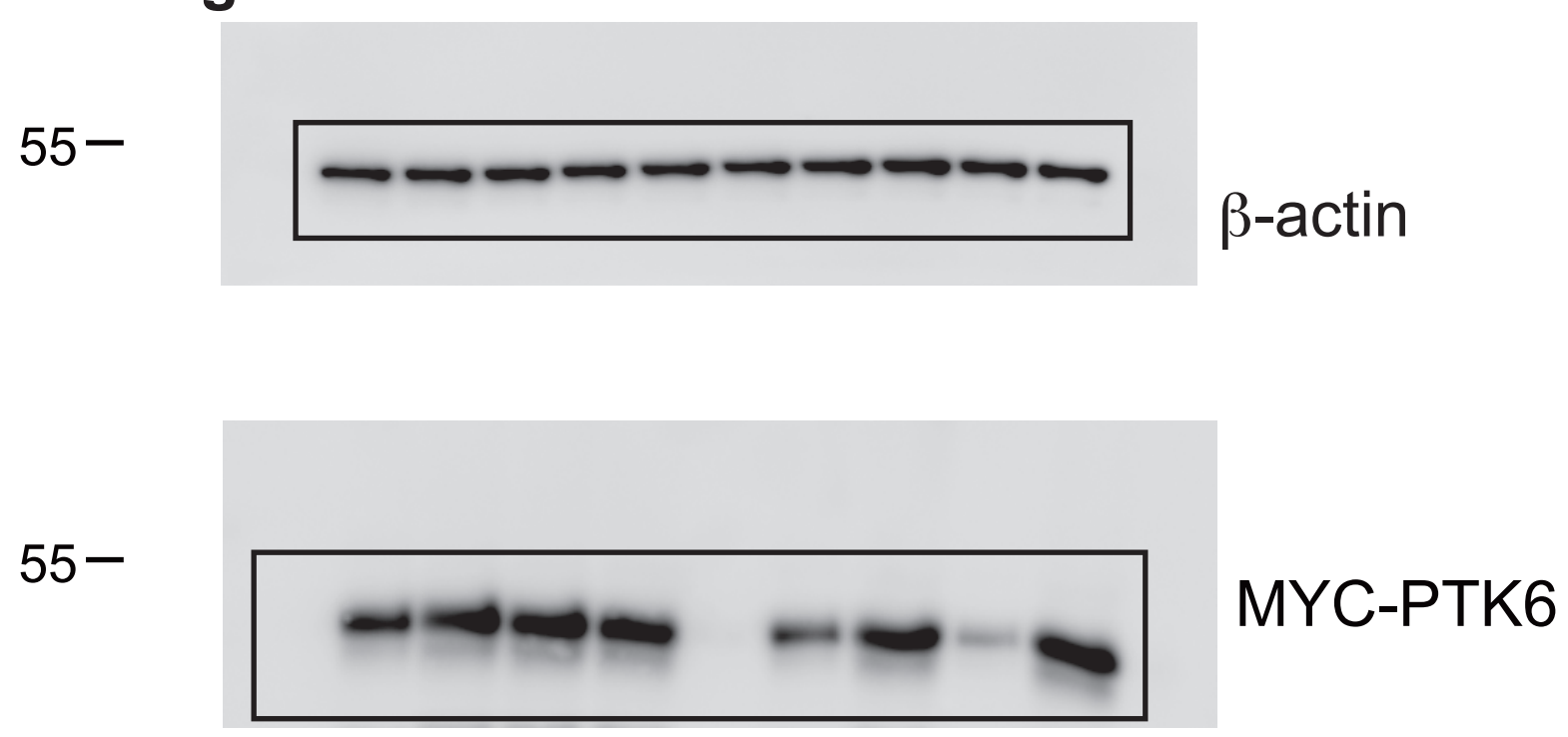

Fig 4C

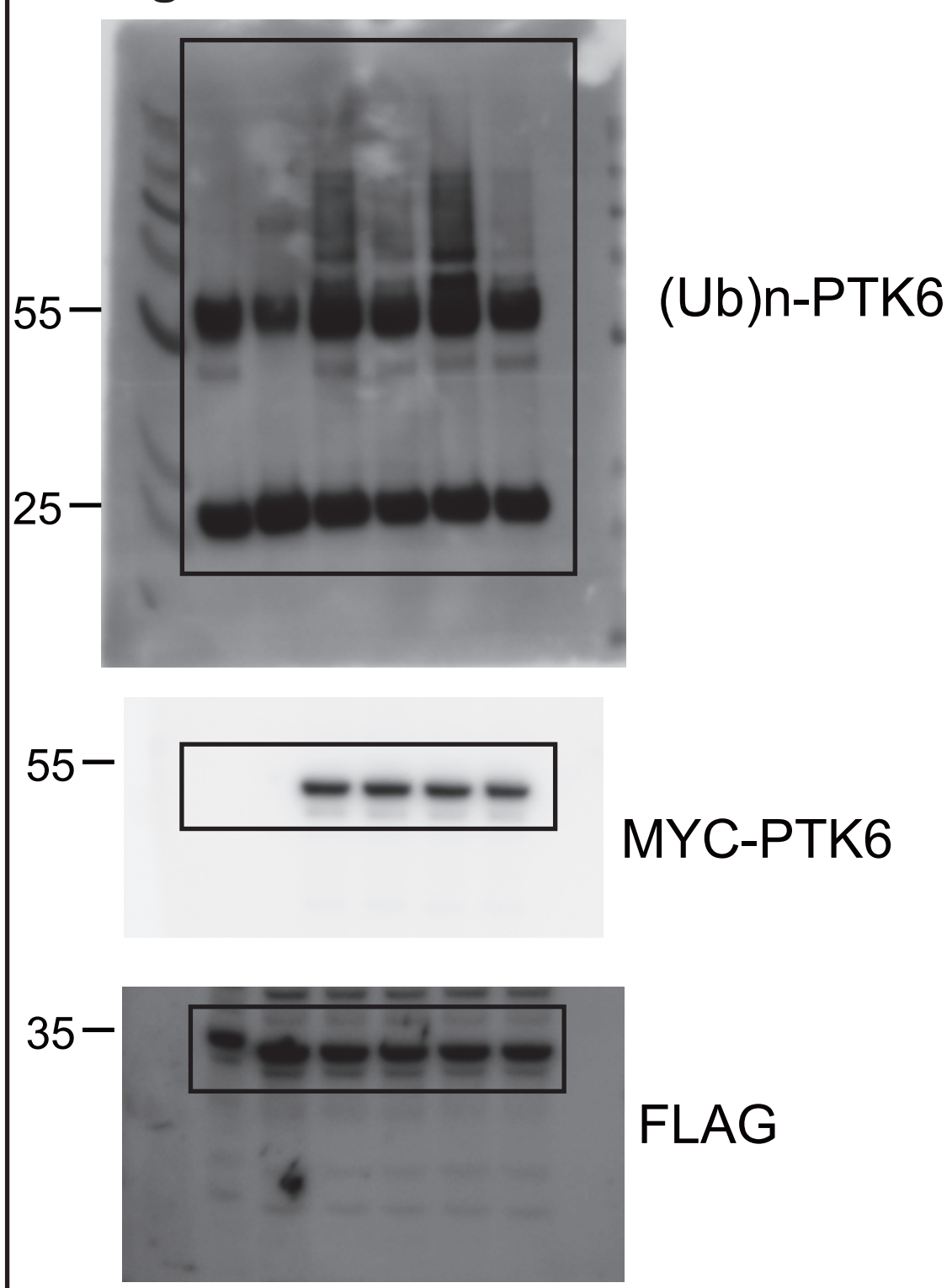

Fig 4D

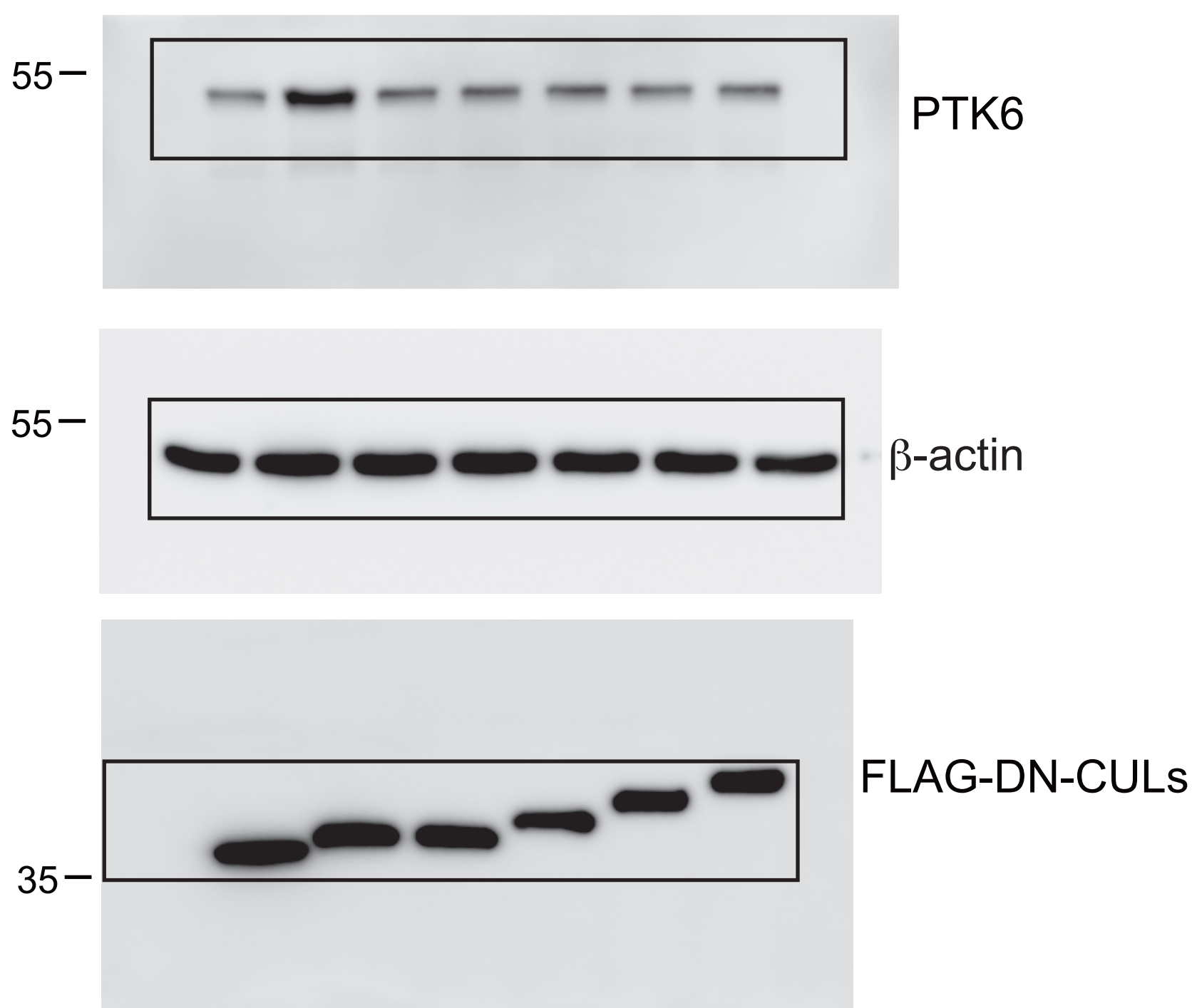

Fig 4E

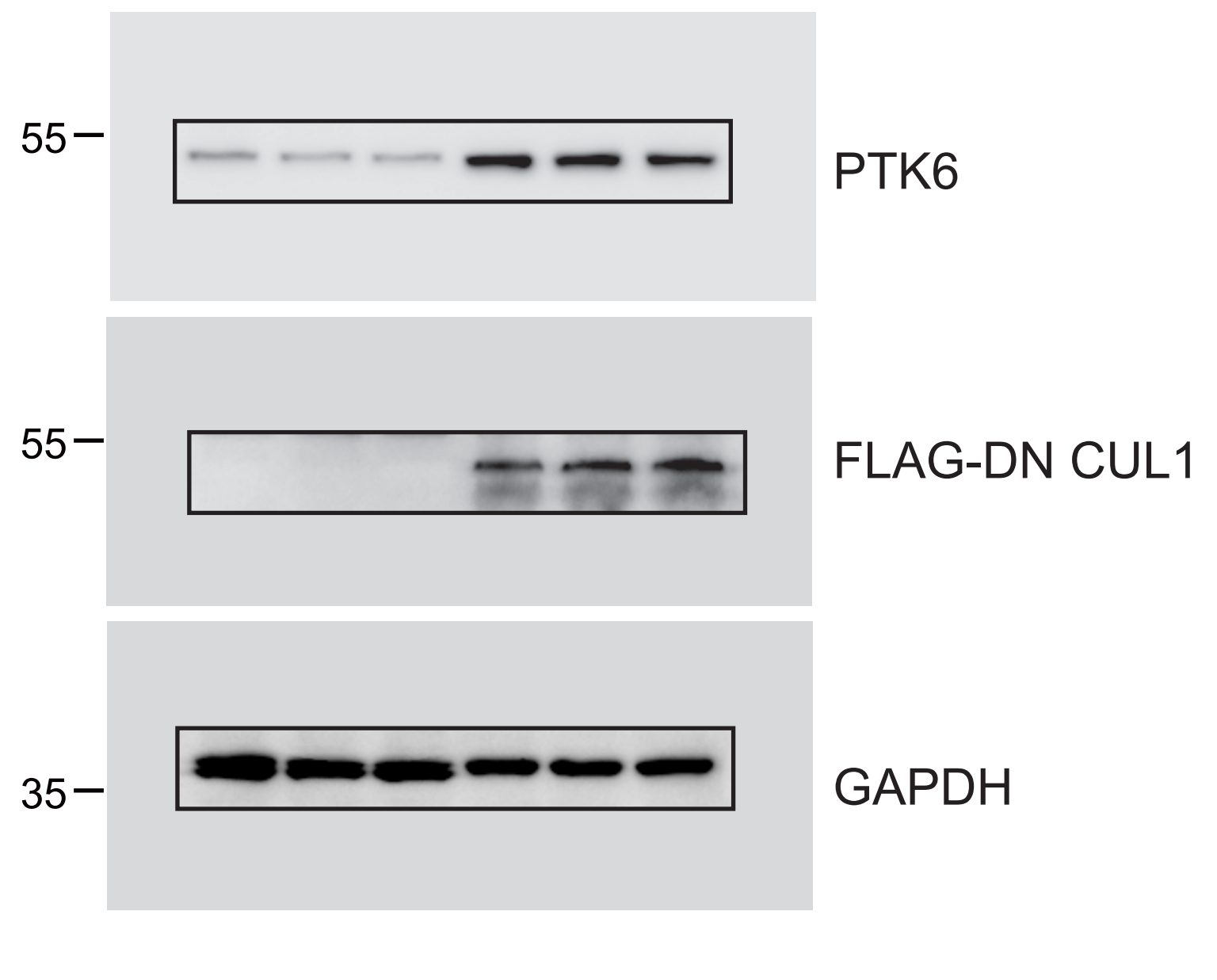

Fig 4F

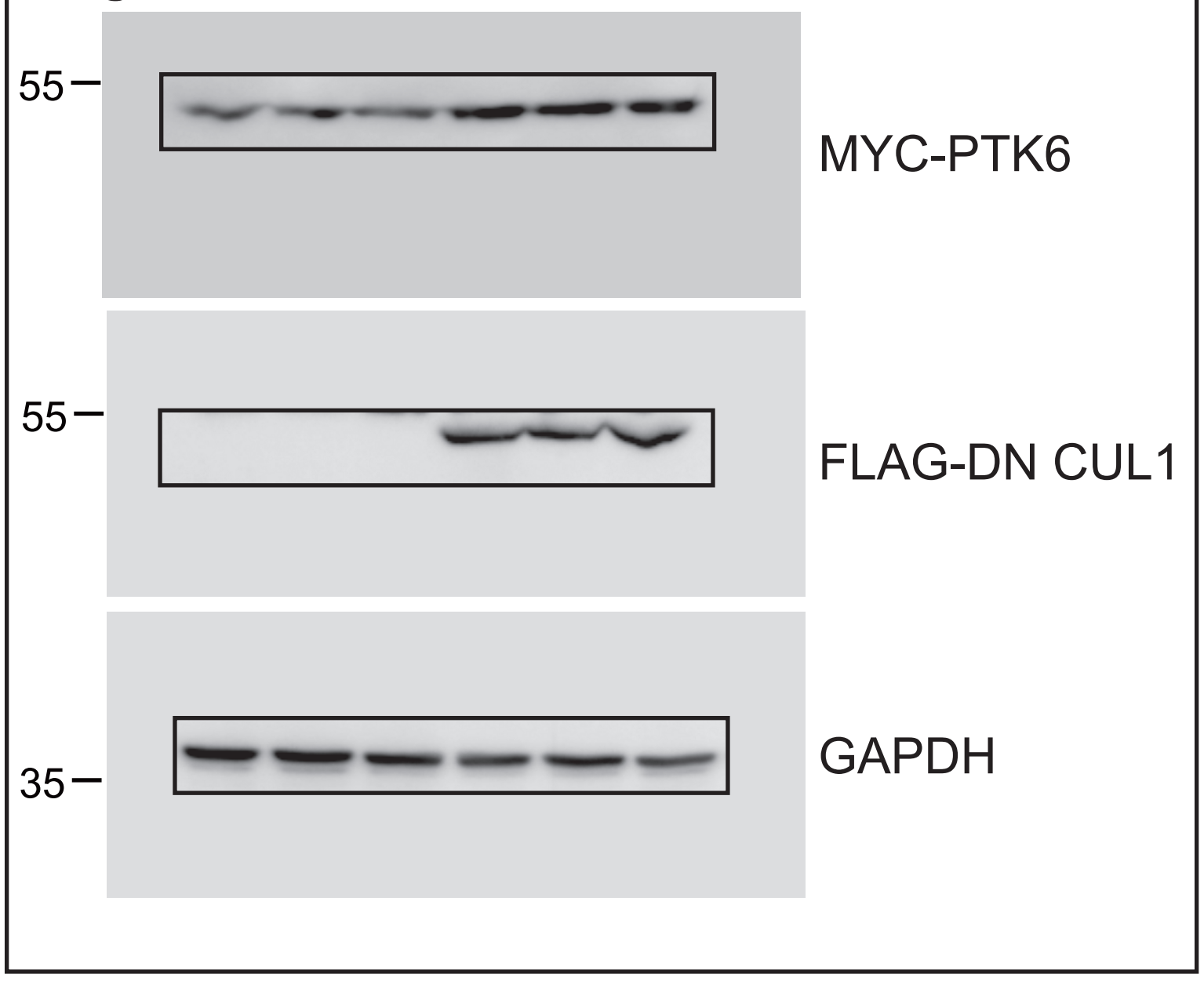

Fig 4G

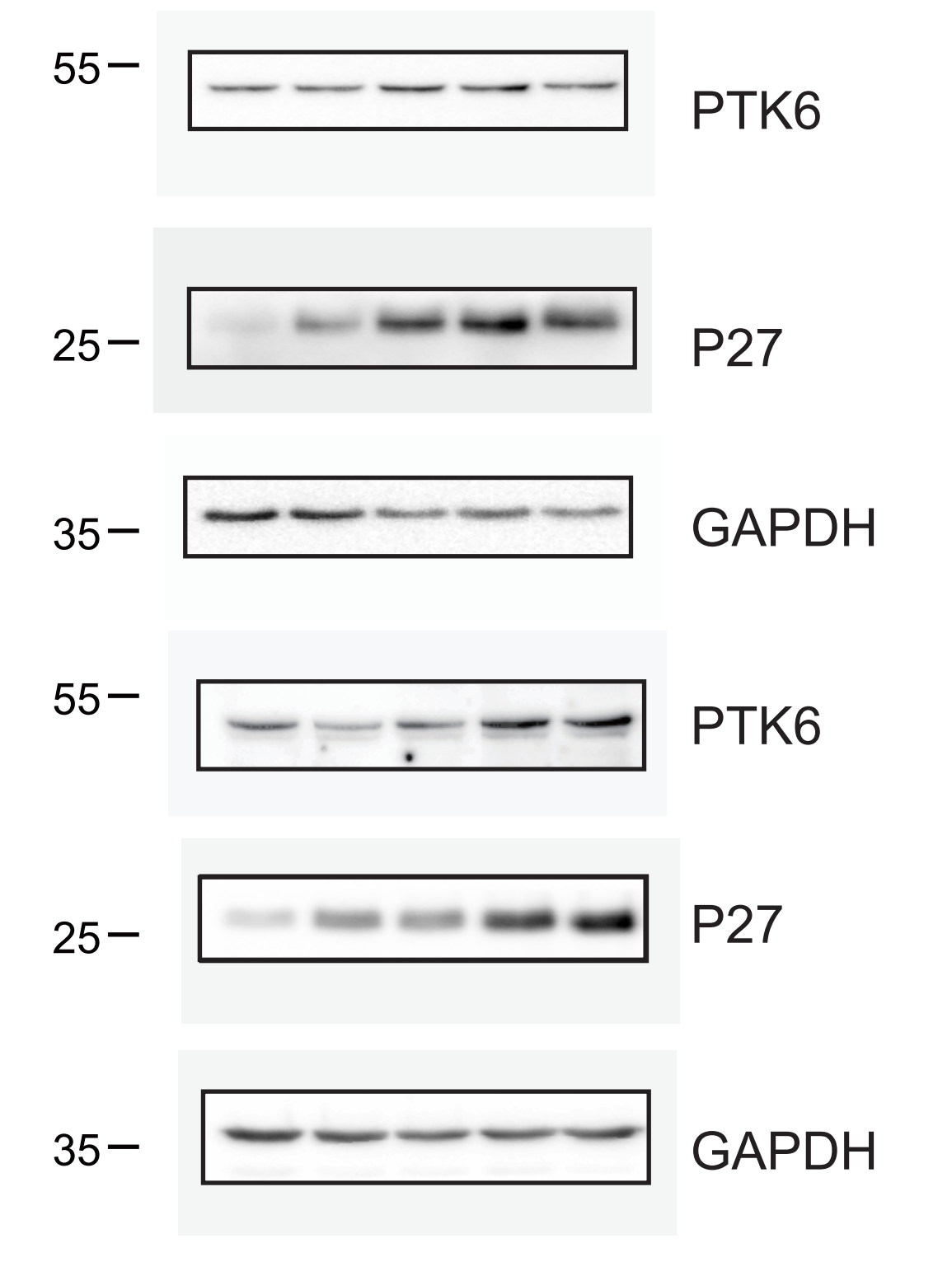

Fig 4H

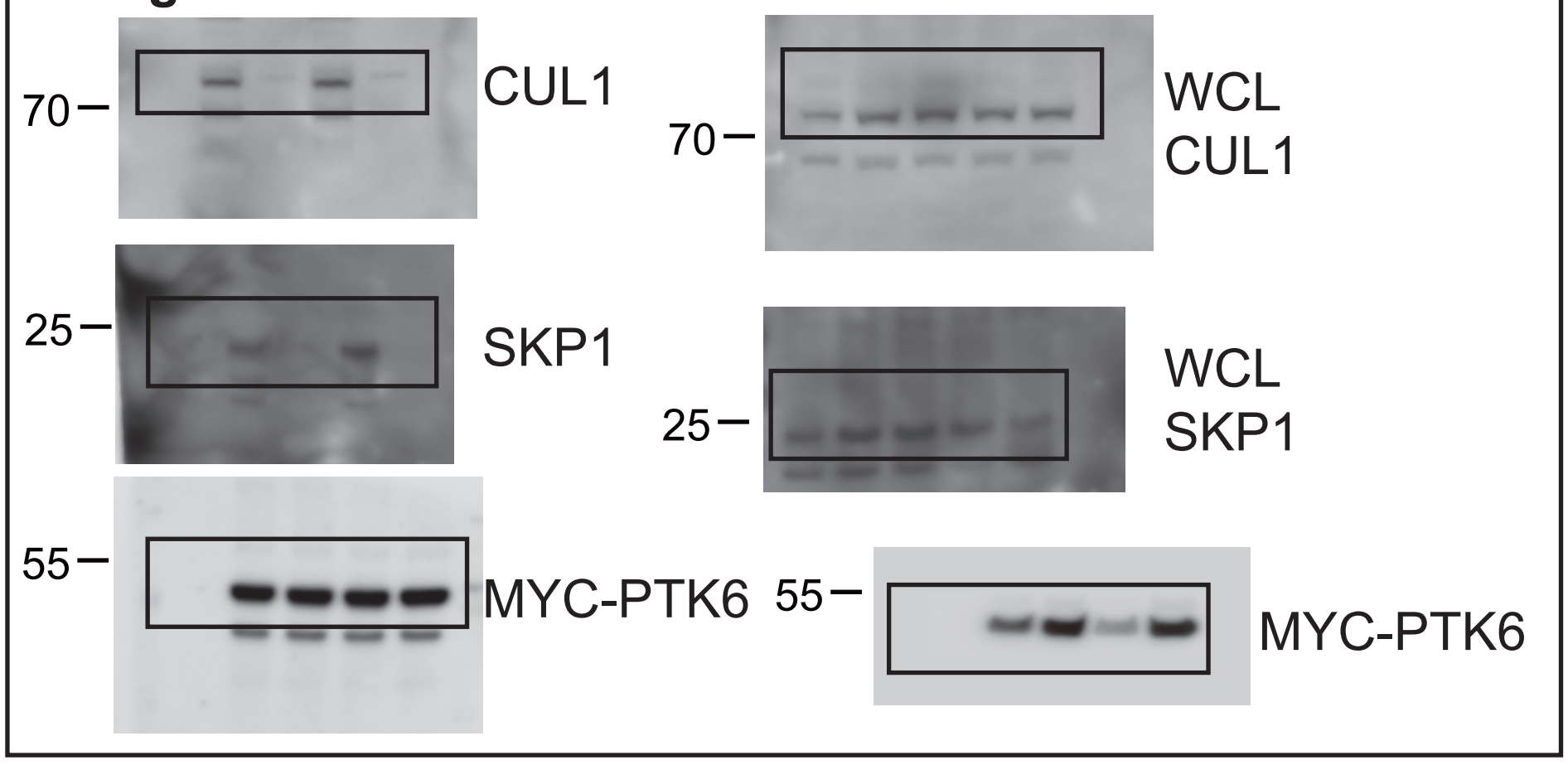

Fig 5A

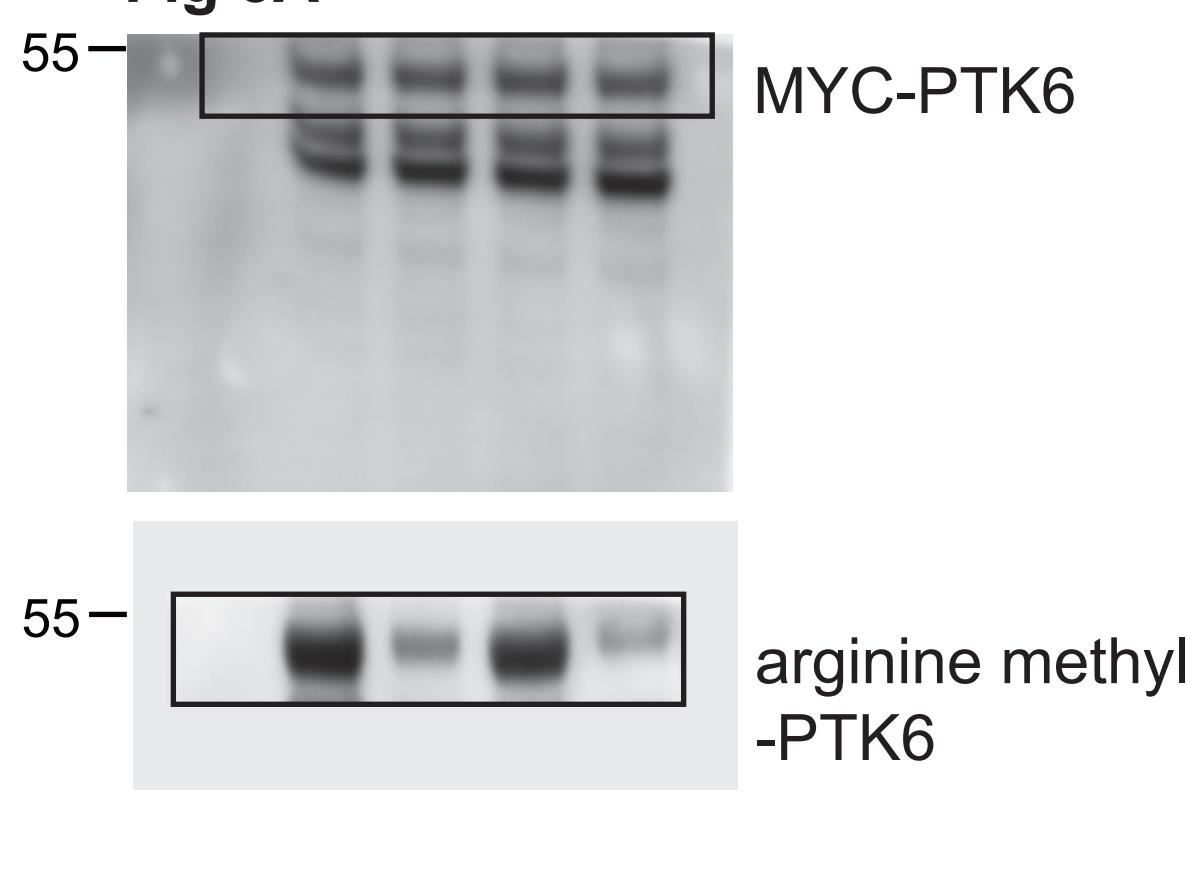

Fig 5B

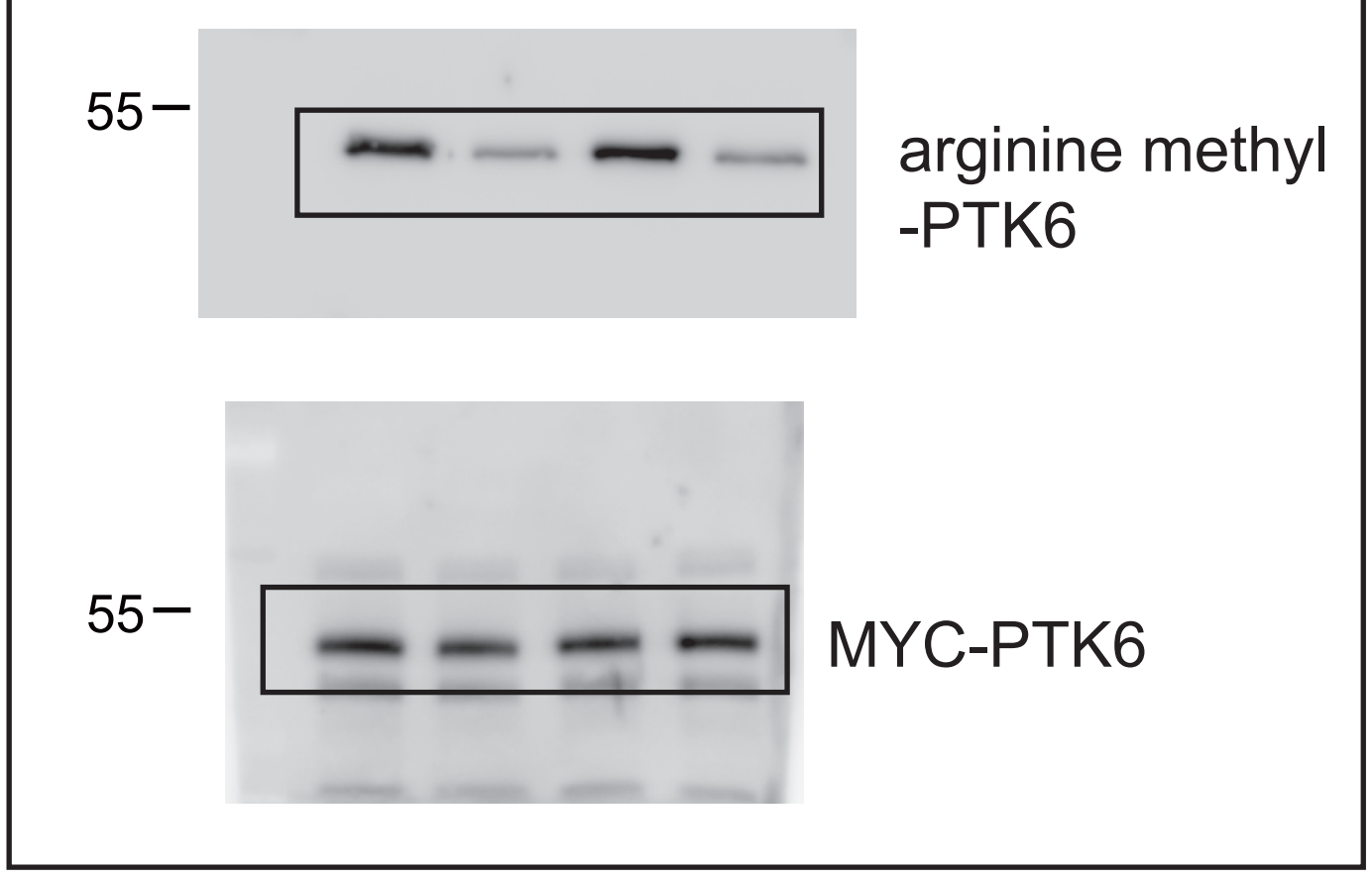

Fig 5C

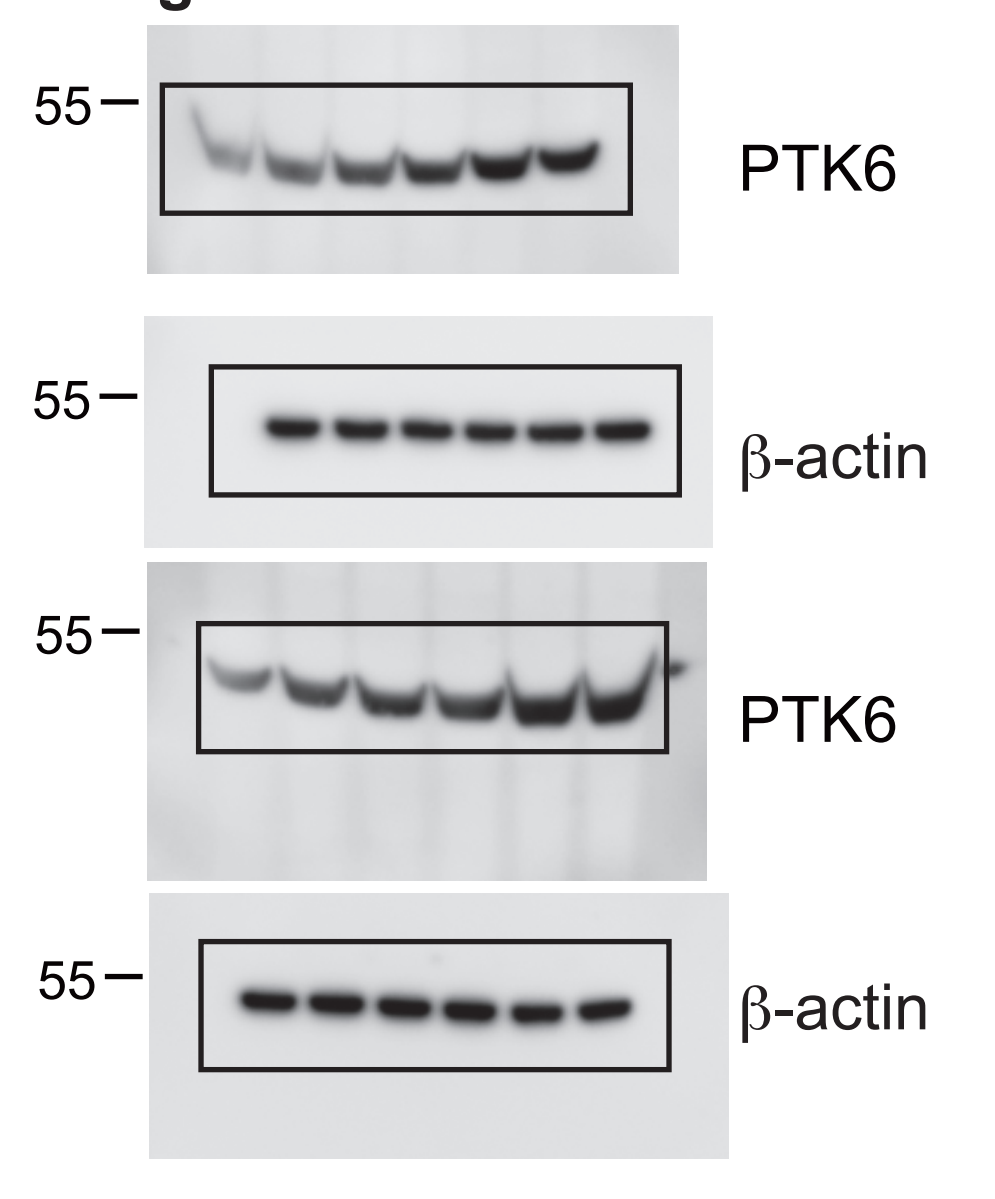

Fig 5D

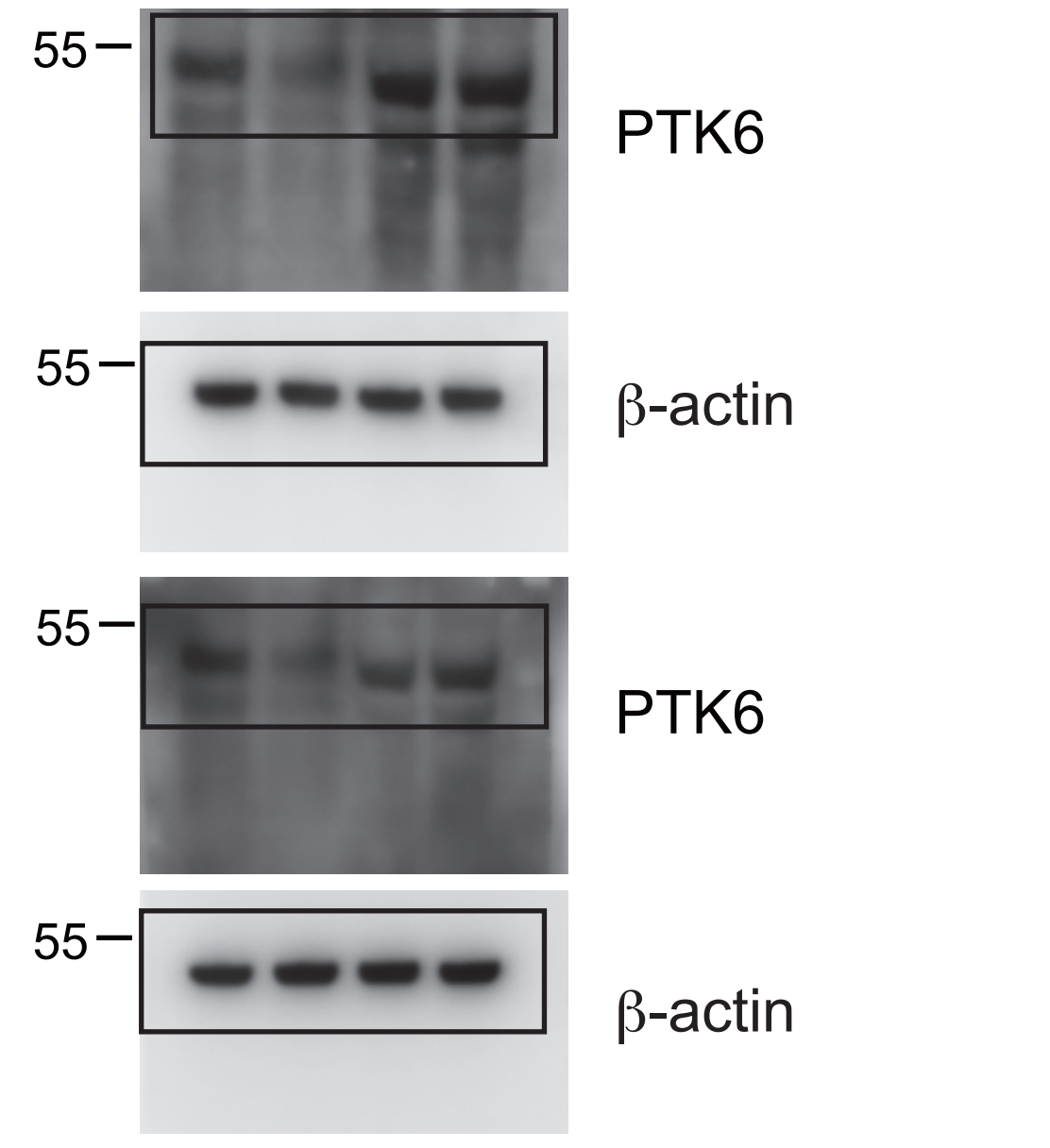

Fig 5E

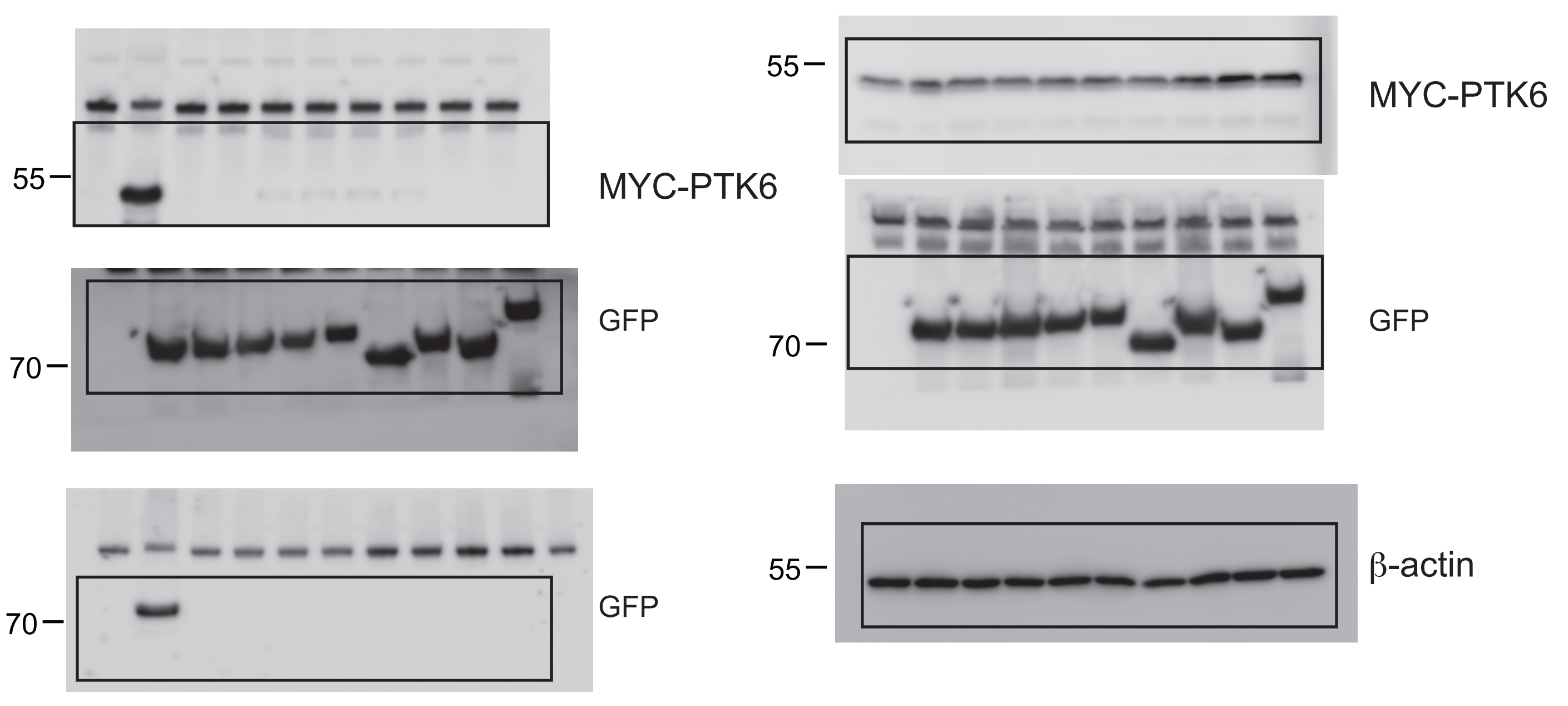

Fig 5F

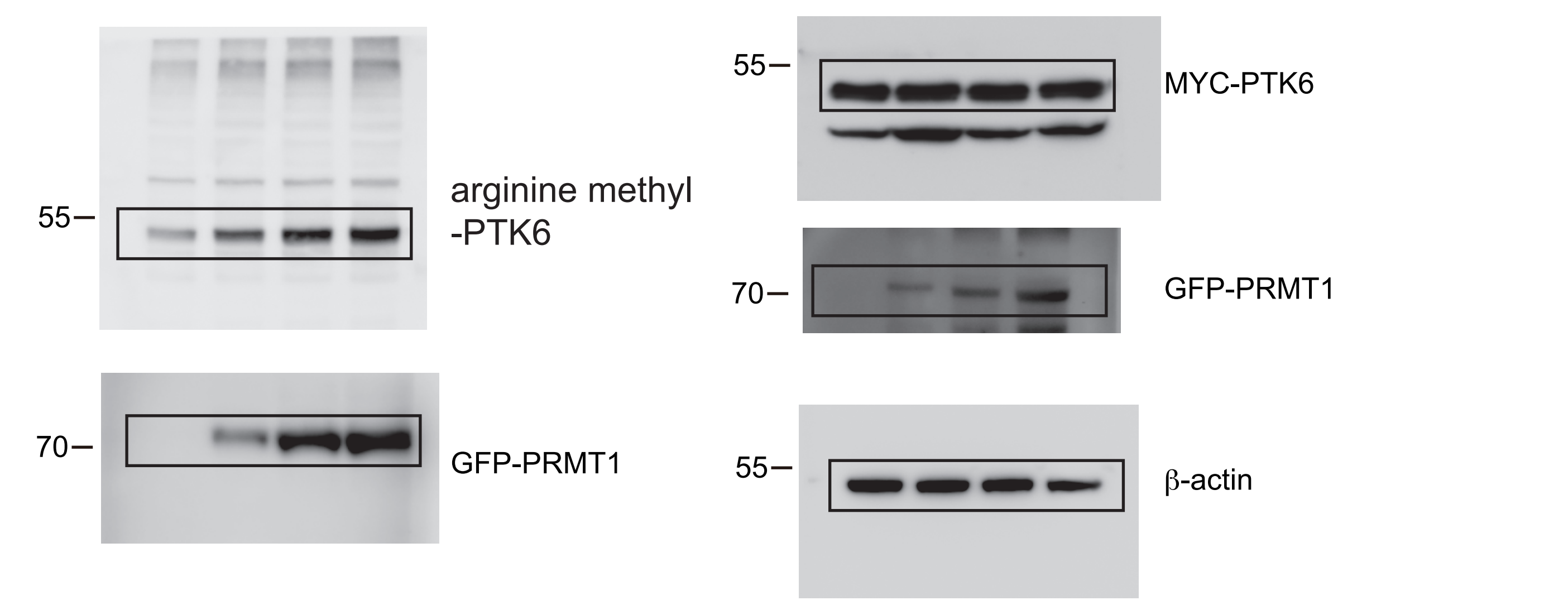

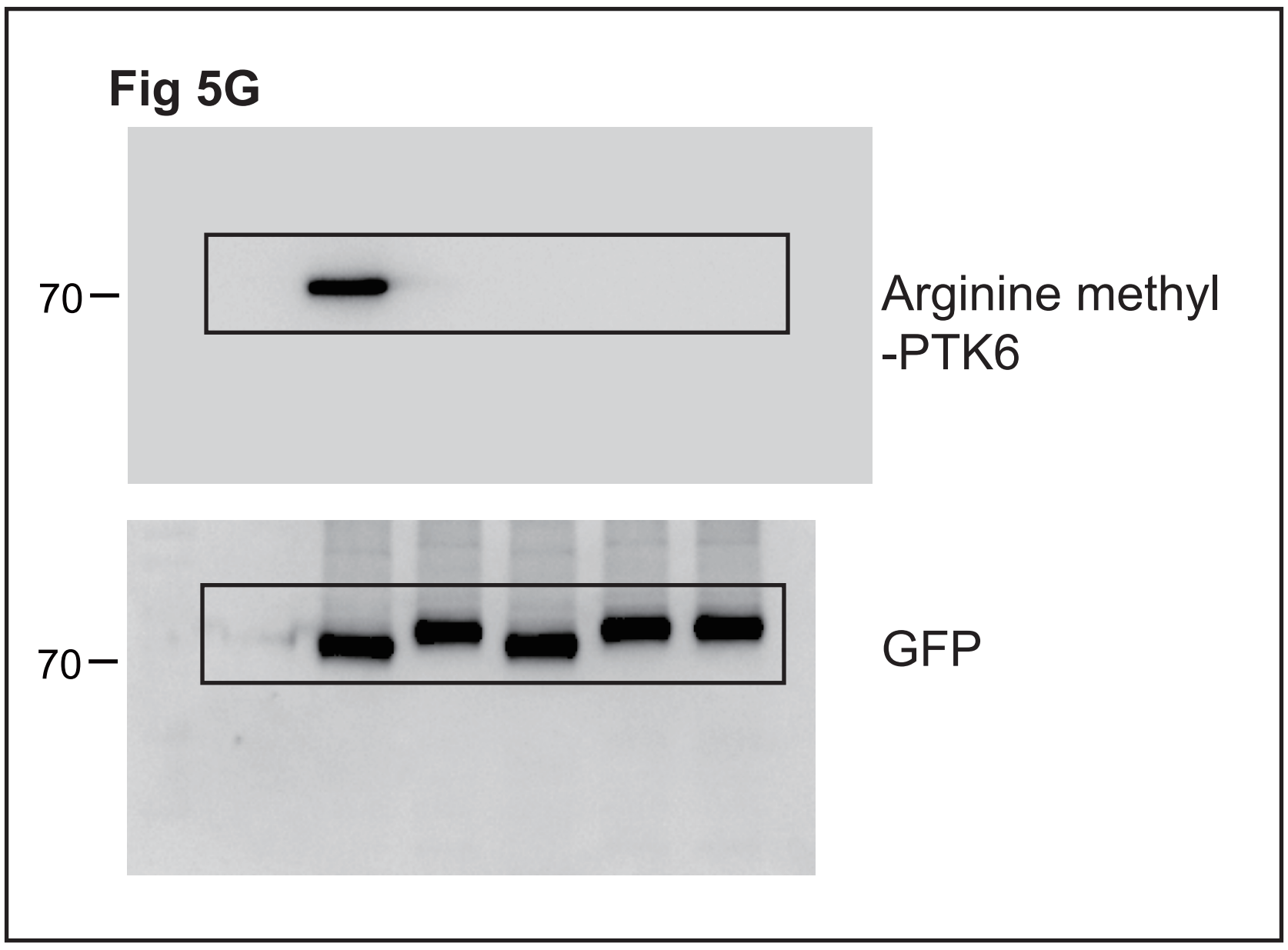

Fig 5G
70
Arginine methyl
-PTK6
70
GFP

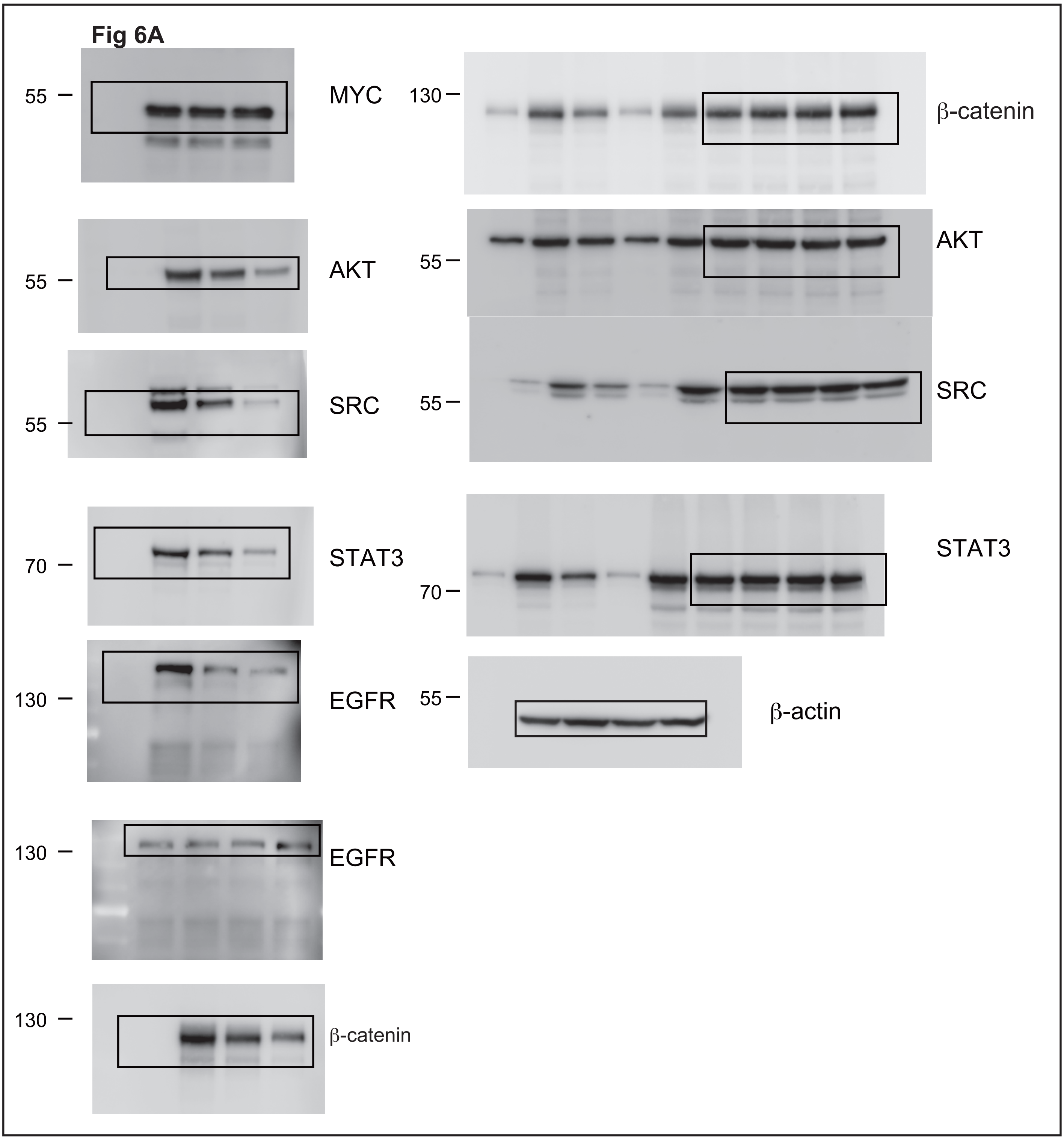

Fig 6A
55
MYC
130
β-catenin
AKT
55
55
AKT
SRC
55
55
SRC
STAT3
70
STAT3
70
EGFR
130
55
β-actin
130
EGFR
130
β-catenin

Fig 6C

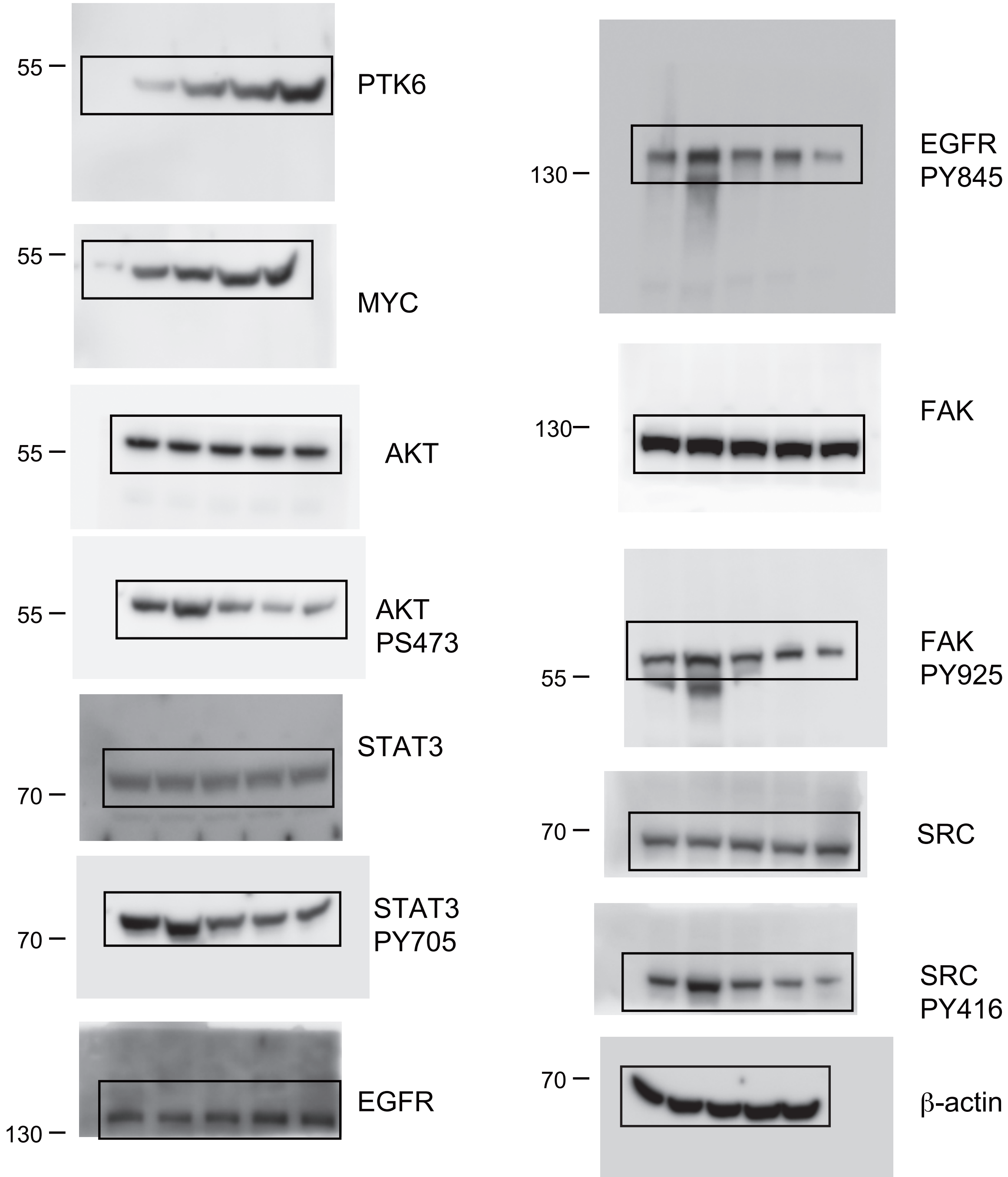

Fig S4A

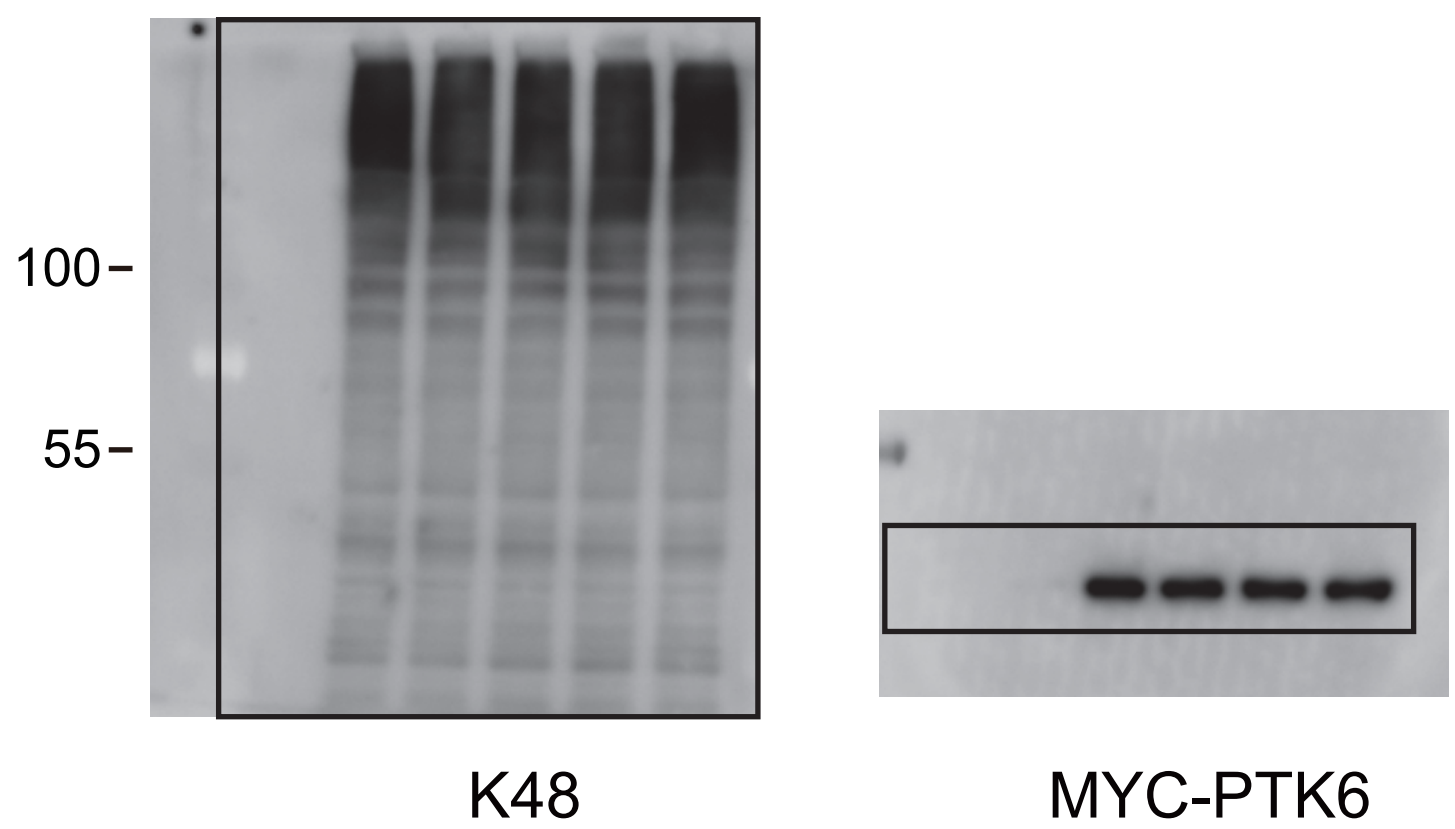

Fig 6E

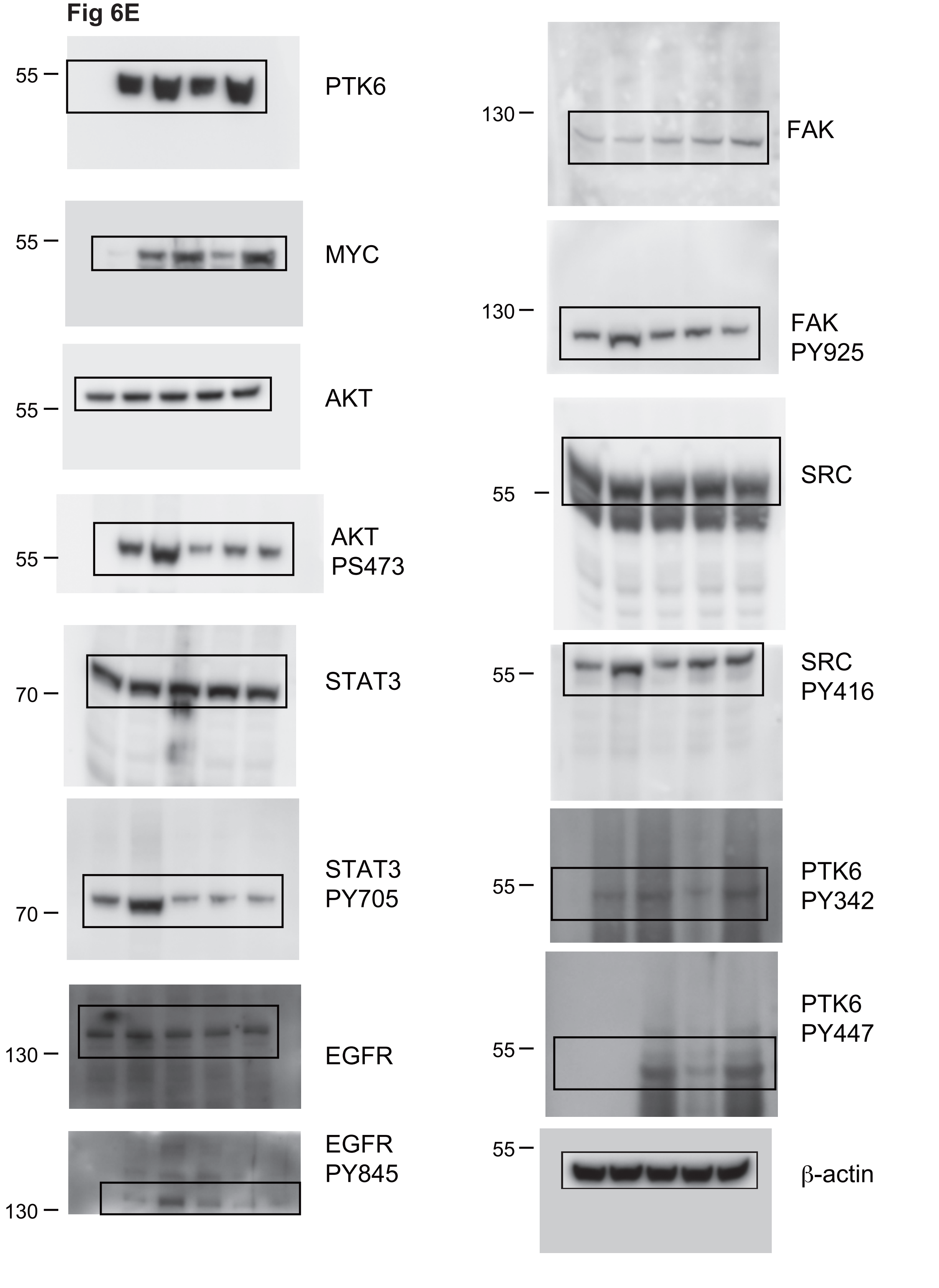

Fig S1C

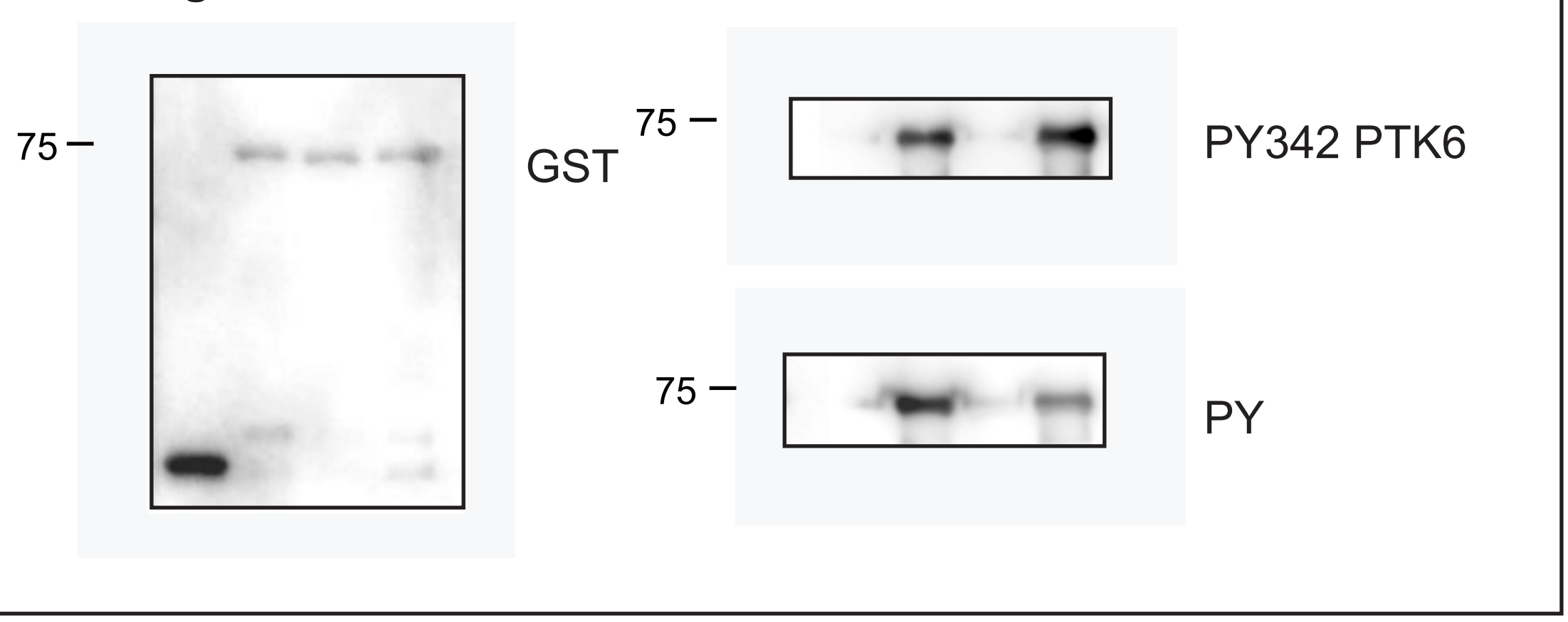

Supplement: Supplementary file 1 [file 41419_2026_8933_MOESM2_ESM.pdf]
