# Supplementary material for: Protein tyrosine kinase-6 is post-translationally regulated by arginine methylation and ubiquitination
Source: Cell Death Dis. 2026 Jun 5;17(1):706. doi: 10.1038/s41419-026-08933-5 (PMC13458159; doi:10.1038/s41419-026-08933-5)
Supplement: Supplementary file 2 [file 41419_2026_8933_MOESM1_ESM.docx]

**SUPPLEMENTAL MATERIAL**

**Protein Tyrosine Kinase-6 is Post-translationally Regulated by Arginine Methylation and Ubiquitination**

Liping Jiang, Milica B. Gilic, Katarina Vlajic, Wenjun Bie, Shafi Kuchay, and Angela L. Tyner

**Supplemental Methods**

**Bacterial protein expression and purification**

GST-tagged human PTK6 WT, catalytically dead PTK6 KM, constitutively active PTK6 YF, and PTK6 SH2 WT, SH2 R131A/R136A and SH2 H126A, were cloned into pGEX-2T using the BamHI and EcoRI restriction sites. Human full length PTK6 WT, KM, YF proteins, as well as the PTK6 domains (SH2 WT, SH2 R131A/R136A and SH2 H126A) were expressed as GST fusion proteins in Novagen Rosetta(DE3)pLysS bacterial strain (Millipore Sigma, Burlington, MA) at 16 degrees in the presence of 1 mM IPTG for 16 h. Proteins were purified using glutathione Sepharose beads in elution buffer (20 mM TRIS, 150 mM NaCl, protease inhibitors, 50 mM glutathione, pH 8.0) and concentrated using Vivaspin columns (Millipore Sigma). The membranes were incubated with 2 pmol of purified proteins in 5 ml of 3% fatty acid free BSA. PIP strips were extensively washed with TBST and lipid-protein interactions were detected using anti-GST antibody.

**Quantitative RT-PCR**

Total RNA was extracted using the RNeasy Mini Kit (Qiagen) and first-strand cDNA was synthesized from 1 µg of total RNA using the iScript™ cDNA Synthesis Kit (Bio-Rad, CA). Quantitative real-time PCR (RT-qPCR) was performed using the DyNAmo™ HS SYBR® Green qPCR Kit (Thermo Fisher Scientific). All reactions were run in triplicate and amplified using the Bio-Rad CFX Duet Real-Time PCR System. Relative gene expression levels were calculated using the 2^–ΔΔCt^ method, with normalization to an internal reference gene β-actin. The following primers were used: human PTK6: (F) 5'-GCTATGTGCCCCACAACTACC-3' and (R) 5'-CCTGCAGAGCGTGAACTCC-3'. Human β-actin: (F) 5'-CATGTACGTTGCTATCCAGGC-3' and (R) 5'-CTCCTTAATGTCACGCACGAT-3'.

**Supplemental Figure Legends**

**Figure S1. Lipid binding experiments using purified bacterially expressed recombinant proteins. A.** Coomassie stained SDS-PAGE of the soluble and flow-through collected from the Glutathione Sepharose column as well as washed, and eluted protein fractions. **B.** SDS-PAGE of purified GST, and GST-tagged PTK6-SH2 WT, PTK6-SH2 R131A/R136A, and PTK6-SH2 H126A before and after Vivaspin 500 column. **C.** The purified proteins were analyzed by immunoblotting for GST. Activity of purified PTK6 WT, PTK6 KM, and PTK6 YF was analyzed using antibodies against active PTK6 (PY342) and total phosphotyrosine (PY). PTK6 WT and YF have activity in bacteria and are capable of autophosphorylation at Y342, and PTK6 WT at Y447. **D.** Lipid dot assays were performed on PIP strips to determine the binding affinity of bacterially expressed full length PTK6 wild type and PTK6 KM. The membranes were incubated with 2 pmol of GST-tagged proteins, and protein binding was detected using GST antibodies. GST was used as a negative control and PI(4,5)P2 Grip as a positive control. **E.** Purified WT and mutant SH2 domains were used in the protein-lipid overlay assay.

**Figure S2. PTK6 activity does not have an impact on lipid binding. A.** Purified GST-tagged PTK6 WT, PTK6 KM and PTK6 YF were incubated with PIP strips and binding was detected using GST antibody. Purified GST protein was used as a control.

**Figure S3. Quantification of the confocal localization of PTK6 mutants in BPH1, LNCaP, DU-145 and PC3 cells.** Colocalization of MYC-tagged PTK6 proteins (green) and the plasma membrane marker PMV-SeQ (red) was quantified by generating intensity profiles along cross-sections (red lines) on confocal images (Figure 2A) using Zen Software (Zeiss, Jena, Germany). AU, arbitrary units.

**Figure S4. A.** **K48-linked polyubiquitinated proteins are enriched by TR-TUBE but not by the TR-TUBE mutant.** The same immunoprecipitated samples shown in Figure 4C were subjected to immunoblotting with K48 polyubiquitin or MYC-tagged PTK6 antibody. PTK6-specific ubiquitination is shown in Fig. 4C by immunoblotting for MYC-PTK6. **B, C**. **Levels of *PTK6* mRNA expression do not change in cells treated with MLN4924 or GSK3368715.** LNCaP and BPH1 cells were treated with 10 μM (1x) MLN4924 for 0, 2, 4, 6, 8 hours and RNA was isolated and subjected to quantitative RT-PCR **(B)**. LNCaP and BPH1 cells were treated with different doses of GSK3368715 as indicated and RNA was isolated at 24 or 48 hours post treatment followed by quantitative RT-PCR **(C)**. No significant differences in PTK6 RNA levels were detected before and after treatment, analyzed by one-way ANOVA followed by Tukey's test (**B**), or paired two-way ANOVA with multiple comparisons (**C)**.
